# A nanocrystal-based PN junction model for quantum dot light-emitting diodes

**DOI:** 10.1038/s41377-026-02356-9

**Published:** 2026-07-17

**Authors:** Hui Bao, Seyed Mehdi Sattari-Esfahlan, Haizheng Zhong

**Affiliations:** MIIT Key Laboratory for Low-Dimensional Quantum Structure and Devices, School of Materials Science & Engineering, Beijing Institute of Technology, Beijing, 100081 China

**Keywords:** Photonic devices, Inorganic LEDs

## Abstract

The PN junction of semiconductors plays an important role in designing and developing light-emitting diodes. In contrast, the characteristics of PN junctions in quantum dot light-emitting diodes (QLED) have been less discussed. In this work, we analyzed the current-voltage (I–V) characteristics of QLED using a modified nano-PN junction model, which combines a silicon-based PN junction model and a hopping transport model. Under the assumption of recombination current dominance in high-efficiency QLEDs, the correlations between complete QLED I–V curves and their constituent sub-device characteristics were derived. The voltage distribution and Quasi-Fermi level splitting of functional layers were performed to elucidate the high ideality factors of QLED devices. The nanocrystal-based PN junction model was extended to simulate the experimental I–V curves of efficient QLED devices. In all, this work not only deepens the device understanding into QLED from the view of semiconductor physics, but also builds up a theoretical framework of nanocrystal-based PN junctions.

## Abstract (web summary)

By developing a modified nano-PN junction model, we can correlate the current-voltage curves of QLED with the corresponding sub-devices of functional layers.

## Graphical abstract

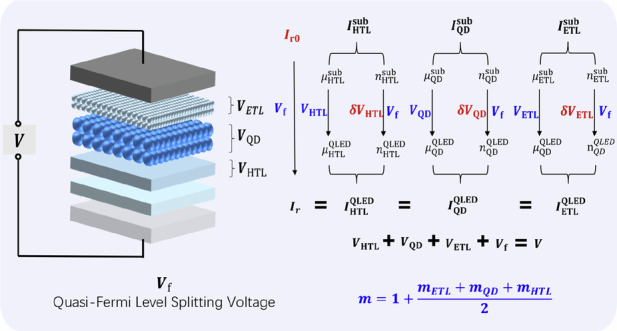

## Introduction

With the development of nanoscience and nanotechnology, quantum dots (QDs) have been successfully applied to the development of solution-processed electronic and optoelectronic devices^1–3^. Especially, the efficiencies of QDs based LEDs^4^, solar cells^5^ and infrared photodetectors^6^ approach to the requirements of industrial applications. Among them, quantum dot light-emitting diodes (QLEDs) are regarded as one of the most promising display technologies^7–9^, owing to their wide color gamut, high brightness, and easy high resolution^10^. Over the past two decades, the research of QLEDs has witnessed considerable advancements in materials^11–14^, efficiency and lifetime^11,13,15,16^, models and device physics^15,17–23^, and fabrication processes^24–27^. However, it still lacks a comprehensive understanding of devices from a semiconductor physics perspective.

PN junction is the basis of semiconductor devices^28,29^, particularly in guiding the design and development of semiconductor devices such as transistors, detector^30^, solar cells^31^, and light-emitting diodes. According to the theory of PN junction, the I–V curves of a typical diode can be fitted using the Shockley equation of $I={I}_{0}\left({e}^{\frac{q{\rm{V}}}{{nkT}}}-1\right)$, where *I*_0_, *q*, *n*, *k*, and *T* represent dark saturation current, elementary Charge, ideality factor, Boltzmann constant and temperature, respectively. Among these parameters, the ideality factor can describe the characteristics of diodes. For instance, it has been successfully applied to analyze the gallium nitride-based LEDs^32^, illustrating the effects of leakage current, ABC recombination model, and ideality factor of recombination current^33–35^. Although the I–V curves of efficient QLED can be fitted using the Shockley equation, it is very difficult to understand the serious deviation of the ideality factor under high voltage (ideal value: 1–2, GaN-LED: 2–6^33^, QLED: >20^36,37^).

To clarify the origin of the high ideality factor in QLED devices, we consider the PN junction of conventional semiconductors and the hopping transport models of disordered semiconductors^38–40^, and develop a nanocrystal-based PN junction model for describing the characteristics of QLED. This model correlates the I–V characteristics of efficient QLED devices with the I–V curves of TFB, QD and ZMO based sub devices. Based on the model, the fitting of these I–V curves illustrate the serious deviation of ideality factor, which well describes the experimental results. In addition, a numerical simulation method is also developed to derive the voltage division across TFB, QD, and ZMO layers with increasing current.

## Results

**Fig. 1:**
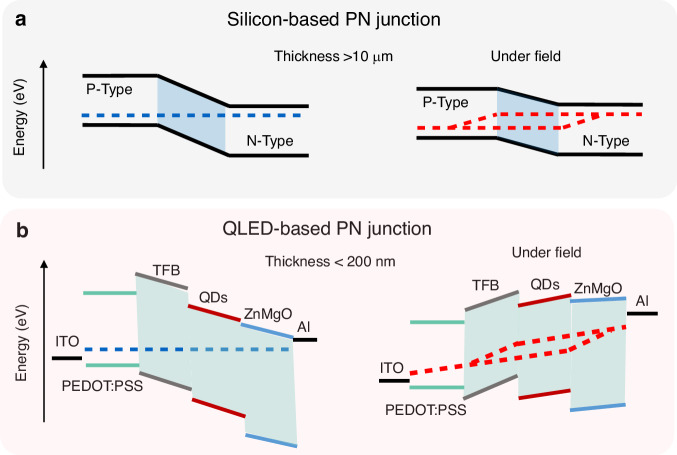
Energy band diagrams of silicon based PN junctions and QLED. **a** Silicon PN junction at equilibrium (zero applied voltage) and under applied voltage. **b** Quantum dot light-emitting diodes (QLED) at equilibrium and under applied voltage

Figure 1a illustrates the energy band diagrams of a typical silicon PN junction. In a silicon-based PN junction, the carrier concentration gradient between p-type and n-type materials drives the formation of a depletion layer and an intrinsic built-in electric field. Due to the ultra-low carrier concentration and high resistivity of the depletion layer, the applied voltage mainly drops across the potential barrier region. Thus, the carrier concentration in the barrier increases with voltage, where the current-voltage relationship is governed by the Shockley equation: $I={I}_{0}\left({e}^{\frac{q{\rm{V}}}{{nkT}}}-1\right)$. In comparison with silicon-based PN junctions, QLEDs are multilayer devices that are composed of disordered semiconductor materials. The charge transport of these function layers follows a hopping transport model with relatively low electrical conductivity^38–40^. Furthermore, the device structure of QLEDs has been revolutionized to improve the electron-hole recombination within the QD emissive layer (Fig. 1b). Based on the intrinsic properties of nanocrystals and structural features of QLEDs, the I–V curves of individual functional layers based on the sub-device were correlated with full QLED devices.

**Fig. 2:**
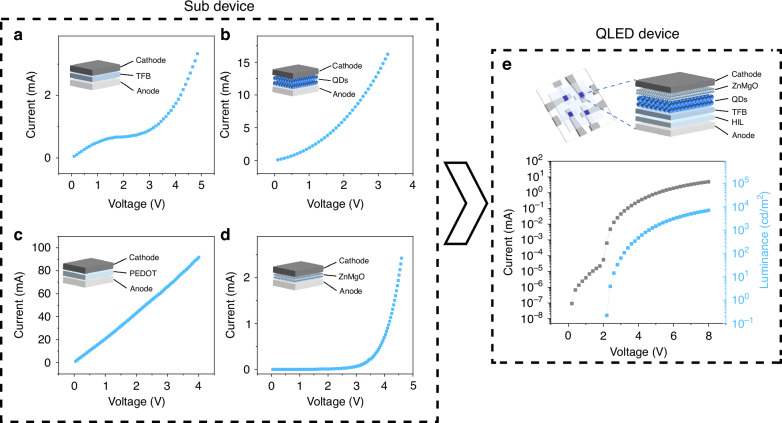
Current-voltage (I–V) characteristics of sub devices and corresponding QLED devices. Single-layer sub devices: **a** ITO/TFB/Al. **b** ITO/QDs/Al. **c** ITO/ PEDOT:PSS/Al. **d** ITO/ZMO/Al, and **e** corresponding QLED devices

A typical QLED structure of ITO/PEDOT:PSS/TFB/QD/ZMO/Al can be split into four sub-devices for investigation, including ITO/PEDOT:PSS/Al, ITO/TFB/Al, ITO/QDs/Al, and ITO/ZMO/Al. Figure 2a–d show the I–V curves of these devices. Linear I–V curves were observed for the sub-devices of ITO/PEDOT:PSS/Al. The conductivity of these ITO/PEDOT:PSS/Al is in good agreement with the sub-devices of ITO/Al, indicating the nearly zero resistance of the PEDOT:PSS layer. This leads us to exclude the consideration of the PEDOT:PSS layer during the analysis of the I–V curves. Notably, the I–V curves of typical sub-devices are non-linear under reverse voltage (Fig. S1a), and the photo responses of these sub-devices under illumination are negligible (Fig. S1b). These phenomena suggest that the I–V characteristics of sub-devices primarily originate from intrinsic material properties, with negligible contributions from interfacial Schottky barriers.

Given that the QD, TFB, and ZMO materials are semiconductors characterized by short-range order and long-range disorder, the carrier transport of these materials is governed by the hopping transport model^39^, in which the carrier tunneling probability (*Γ*) between adjacent states is defined as:

1 $$\varGamma \propto {e}^{-\frac{2R}{\alpha }-\frac{W}{{kT}}}$$

Here *α* represent the localization length, *W* and *R* represent the energy mismatch and distance between the donor and acceptor site. Previous works has derived several theoretical models, including variable-range hopping (VRH) and nearest-neighbor hopping (NNH)^40^, which can be clarified by applying temperature-dependent conductivity measurements. There are already a few of established equations for describing the I–V curves of disordered semiconductors. For example, in the work of A.V. Dvurechenskii et al.^41^ an expression for the electric field-dependent conductivity of amorphous silicon was proposed as: ${\rm{\sigma }}\left({\rm{E}}\right)\,\propto {e}^{-{(\frac{{E}_{0}}{E})}^{0.5}}$. Meanwhile, in the review by Bassler et al.^42^ the electric field dependence of carrier mobility was summarized as: ${\rm{\mu }}\left({\rm{E}}\right)\propto {{\mu }_{0}e}^{{(\frac{E}{E0})}^{0.5}}$. Similar works by Pasveer et al.^43^ derive the expression for the electric field-dependent mobility with the formula: ${\rm{\mu }}\left({\rm{E}}\right)\propto {{\mu }_{0}e}^{A(\sqrt{1+B{E}^{2}}-1)}$. Considering the similarity of exponential forms, an empirical formula (${{\rm{V}}}_{d}={A{\rm{e}}}^{\frac{{\rm{V}}}{m{\phi }}}$) was adopted to describe the field-dependent carrier drift velocity, where *A* and *m* are empirical parameters related to material-specific hopping processes and *m* is designated as the characteristic factor of the function layers. ϕ = kT/q represents the thermal voltage. Applying this assumption to disordered semiconductor films, the current of sub-devices can be modeled as:

2 $${I}_{{\rm{k}}0}={n}_{{\rm{k}}0}{qS}{{A}_{{\rm{k}}}{\rm{e}}}^{\frac{{V}_{{\rm{k}}}}{{m}_{{\rm{k}}}{\phi }}}$$

where ${n}_{k0}$, q, and S denote the intrinsic carrier density, elementary charge, and cross-sectional area of layer k (ZMO, QD, or TFB). According to Eq. 2, the nonlinear I–V characteristics of the sub-devices originate from the voltage-dependent drift velocity due to the hopping transport.

We also analyze the I–V characteristics of fabricated QLEDs using the aforementioned thin-film processes (Fig. 2e). The I–V curve is typically divided into two distinct regimes: (i) the pre-turn-on region and (ii) the post-turn-on region. Although there are different explanations concerning the I–V curves in the pre-turn-on stage, it still lacks direct theoretical justification. After the turn-on stage, the typical I–V curve exhibits exponential-like behavior, which is very similar to the Shockley equation for PN junctions. However, the exponential fitting of the I–V curves indicates that the ideality factor (n) of QLED devices is significantly higher than the theoretical values predicted by the Shockley equation for PN junctions (usually between 1 and 2). To illustrate the PN junction characteristics in QLEDs, the formation of PN junctions in QLED and the corresponding operational mechanisms are analyzed.

In silicon-based PN junctions, the carrier diffusion between the p-type semiconductor and n-type semiconductor accounts for the formation of the depletion layer (Fig. 1a). As shown in Fig. S13, the thickness of the depletion layer (*W*) increases from 142 nm to 23 μm as the carrier concentration decreases from 10^17^ cm^−3^ to 10^12^ cm^−3^. Regarding the formation of nanocrystal-based PN junctions in QLED devices. Based on the I–V curves and mobility reported in the literature^44–46^, the carrier concentration of TFB, QD, and ZMO was estimated to be between 10^10^ cm^−3^ and 10^13^ cm^−3^. According to the calculation examples shown in SI, the PN junctions of these materials have a very thick depletion layer with a thickness of 5.61–10.09 μm, which is larger than the thickness of the functional layers in efficient QLEDs. Similarly, the simulations by Pal et al. suggest a depletion width of several hundred nanometers in PbS nanocrystal-based PN junctions, substantially exceeding the thickness of actual functional devices^47^. Therefore, the function layers of QLEDs should be fully depleted (Fig. 1b). Furthermore, owing to the intrinsic discontinuous feature of the functional layer in QLEDs, the carrier behavior is modeled using a fixed Fermi level shift (*qδV*_*k*_) across each layer. The carrier concentration ($\bar{{n}_{{\rm{k}}}}$) of functional layers k in a QLED can be described using the following expression:

3 $$\bar{{n}_{{\rm{k}}}}={n}_{{\rm{k}}0}\,{e}^{\frac{-\delta {V}_{{\rm{k}}}}{{\phi }}}$$

Here, *n*_k0_ and $\bar{{n}_{{\rm{k}}}}$ denote the carrier concentrations before and after PN junction formation, respectively. This formula establishes the carrier concentration change of the function layer (ZMO, QD, or TFB) before and after the formation of PN junctions.

In silicon PN junctions, the applied voltage mainly drops across the depletion region (Fig. 1a). As a result, the carrier concentration in the depletion region increases with rising applied voltage. The voltage drop in the depletion region is denoted as the quasi-Fermi level voltage. In comparison, the nanocrystal-based PN junctions of QLED at least show three key features, including fully depletion, nonlinear I–V characteristics, and poor conductivity. As a result, the applied voltage affects the carrier concentration in the depletion region, and the carrier transport in the functional layer (Fig. 1b). These differences were further refined to four assumptions for describing the PN junction characteristics of QLED. (i) Given that high-efficiency QLEDs have already achieved EQE over 25%, recombination current dominates the I–V curve after the turn-on region. This assumption is also applicable to the QLEDs with low EQE, even though their non-radiative recombination is dominated. (ii) Given the very thin thickness of QLED devices and the low carrier density of the function layers, the functional layer is assumed to be fully depleted. (iii) Given the low conductivity of the functional layers, the voltage drop is divided into two components consisting of distributed voltage across individual functional layers related to field-dependent carrier mobility and quasi-Fermi level splitting voltage related to increasing carrier concentrations, which is analogous to classical silicon-based PN junction behavior. (iv) Given the low carrier density of each function layer, the carrier distribution is assumed to follow the Boltzmann statistics.

When applying a fixed voltage on a silicon PN junction, quasi-Fermi level splitting occurs with carrier concentration increasing (Fig. 1a). In ideal conditions, the splitting between electron and hole quasi-Fermi levels is equal to the product of applied voltage and elementary charge. In the nanocrystal-based PN junction of QLED, the splitting between electron and hole quasi-Fermi levels is less than the product of applied voltage and elementary charge due to the voltage distribution in the function layers. Mathematically, the applied voltage is defined as *V*, the quasi-Fermi level splitting voltage as *V*_f_, the voltage drop across the TFB layer as *V*_TFB_, the voltage drop across the QD layer as *V*_QD_, and the voltage drop across the ZMO layers as *V*_ZMO_, respectively. Meanwhile, the operation of QLED relies on the electron (or hole) transport from the electrode to QD and the subsequent recombination of electron and hole in QDs. Thus, *I*_HTL_, *I*_QD_, *I*_ETL_ and *I*_r_ are defined as the transport current in the TFB layer, transport current in the QD layer, transport current in the ZMO layer, and recombination current in the QD layer, respectively. In addition, the relationships of these values can be described using the following equation.

4 $${V}_{{\rm{HTL}}}+{V}_{{\rm{QD}}}+{V}_{{\rm{ETL}}}+{V}_{{\rm{f}}}=V$$

5 $$I={I}_{{\rm{r}}}={I}_{{\rm{HTL}}}={I}_{{\rm{ETL}}}={I}_{{\rm{QD}}}$$

Accordingly, the electron quasi-Fermi level splitting affects the electron concentration in ZMO and QD, while the hole quasi-Fermi level splitting determines the hole concentration in TFB and QD. To simplify the calculation, it is assumed that electron quasi-Fermi level splitting is equal to hole quasi-Fermi level splitting in the nanocrystal-based PN junction of QLED. The influence of asymmetric quasi-Fermi level splitting is described in the Supporting Information and briefly analyzed in later sections. Therefore, the following is obtained.

6 $$q\left({E}_{{\rm{fn}}}-{E}_{{\rm{f}}}\right)=q\left({E}_{{\rm{f}}}-{E}_{{\rm{fp}}}\right)={\frac{1}{2}V}_{{\rm{f}}}$$

Where *E*_f_, *E*_fn_ and *E*_fp_ represent equilibrium Fermi level, electron quasi-Fermi level and hole quasi-Fermi level, respectively.

Under applied voltage, Eq. 7 describes the electron or hole concentration of the *k*-th function layer (*n*_k_) in an operated QLED.

7 $${n}_{{\rm{k}}}=\bar{{n}_{{\rm{k}}}}{e}^{\frac{q{V}_{{\rm{f}}}}{2{kT}}}={n}_{{\rm{k}}0}{e}^{\frac{{V}_{{\rm{f}}}}{2{\phi }}-\frac{\delta {V}_{{\rm{k}}}}{{\phi }}}$$

Here *k* represents HTL, QD, and ZMO layers, respectively. *n*_k0_ represents average carrier concentrations before PN junction formation. According to Eq. 2, the transport current of each function layer in an operating QLED can be described as:

8 $${I}_{{\rm{k}}}={n}_{{\rm{k}}}{qS}{A}_{{\rm{k}}}\,{{\rm{e}}}^{\frac{{V}_{{\rm{k}}}}{{m}_{{\rm{k}}}{\phi }}}=\bar{{n}_{{\rm{k}}}}{qS}{A}_{{\rm{k}}}\,{{\rm{e}}}^{\frac{{V}_{{\rm{k}}}}{{m}_{{\rm{k}}}{\phi }}+\frac{{V}_{{\rm{f}}}}{2{\phi }}}={n}_{{\rm{k}}0}{qS}{A}_{{\rm{k}}}{{\rm{e}}}^{\frac{{V}_{{\rm{k}}}}{{m}_{{\rm{k}}}{\phi }}+\frac{{V}_{{\rm{f}}}}{2{\phi }}-\frac{\delta {V}_{{\rm{k}}}}{{\phi }}}$$

Here it should be mentioned that both electron and hole transport currents exist in the QD layer. Because of the current continuity in the QD layer, the transport currents of electrons and holes are equal to each other. Therefore, only one of them is considered in the formula derivation. To simplify the calculation, we set ${B}_{{\rm{k}}} = {n}_{k0}{qS}{A}_{{\rm{k}}}{{\rm{e}}}^{-\frac{\delta {V}_{{\rm{k}}}}{{\phi }}}$. Similarly, *k* represents HTL, QD, and ZMO layers, respectively.

Based on the time-resolved electroluminescence (TREL) result^23^, it is assumed that bimolecular recombination dominates in the QD layer. This assumption also aligns with the fact that radiative recombination must dominate in high-efficiency QLED devices. Thus *I*_r_ can be expressed as Eq. 9.

9 $${I}_{{\rm{r}}}={q{r}_{1}n}_{{\rm{QD}}}{p}_{{\rm{QD}}}{V}_{{\rm{m}}}\,=q{r}_{1}{V}_{{\rm{m}}}{n}_{{\rm{i}}}^{2}{e}^{\frac{{V}_{f}}{{\phi }}}$$

Here ${n}_{{\rm{i}}}^{2}$, *r*_1_ and *V*_m_ represent carrier concentration product of QD (cm^−6^), bimolecular recombination coefficient (cm^3^ s^−1^) and QD layer volume (cm^3^), respectively. To simplify the calculation, we set $q{r}_{1}{V}_{{\rm{m}}}{n}_{{\rm{i}}}^{2}={I}_{{\rm{r}}0}$.

According to Eqs. 4, 5, 8, 9, Eq. 10 is obtained.

10 $$I={I}_{{\rm{r}}}=\,q{r}_{1}{V}_{{\rm{m}}}{n}_{{\rm{i}}}^{2}{e}^{\frac{{V}_{{\rm{f}}}}{{\phi }}}={I}_{{\rm{r}}0}{e}^{\frac{V-A}{m{\phi }}}={I}_{{\rm{s}}}{e}^{\frac{V}{m{\phi }}}$$

The above equation can be solved to obtain.

11 $${I}_{{\rm{s}}}={I}_{{\rm{r}}0}{e}^{\frac{-A}{m{\phi }}}$$

12 $$A=\phi \left({m}_{\mathrm{HTL}}ln\frac{{I}_{{\rm{r}}0}}{{B}_{{\rm{H}}\mathrm{TL}}}+{m}_{\mathrm{QD}}ln\frac{{I}_{{\rm{r}}0}}{{B}_{{\rm{Q}}{\rm{D}}}}+{m}_{\mathrm{ETL}}ln\frac{{I}_{{\rm{r}}0}}{{B}_{{\rm{E}}\mathrm{TL}}}\right)$$

13 $$m=\frac{2+{m}_{{\rm{ETL}}}+{m}_{{\rm{QD}}}+{m}_{{\rm{HTL}}}}{2}$$

Equations 10, 11, 12, 13, and 2 established the relationship between the I–V curves of sub-devices and QLED. It is noticed that Eq. 10 is analogous to the Shockley equation, while the main distinction lies in the ideality factor, as described by Eq. 13.

Furthermore, the ideality factors of practical QLED devices with nonradiative recombination or asymmetric quasi-Fermi level splitting were also considered. The details of the derivation are provided in part 1 of the Supplementary Information. For the device with nonradiative recombination, an average recombination order of *l* (usually from 1 to 2) is assumed in the QD layer. In general, a smaller *l* leads to a smaller ideality factor for the same sub-device parameters, which may be related to low EQE. For device with asymmetric quasi-Fermi level splitting, characteristic factors *m*_QDn_ and *m*_QDp_ are assumed for electron and hole hopping processes in the quantum dot layer, respectively. When these characteristic factors differ from each other due to asymmetric injection barriers, asymmetric quasi-Fermi level splitting occurs. Under this condition, the overall device ideality factor can still be described by Eq. 13, while the characteristic factors of QD layer need to be replaced by the harmonic mean of *m*_QDn_ and *m*_QDp_.

According to our model, the difference in ideality factors in QLEDs can be attributed to voltage-dependent carrier concentration due to the voltage division modulation of the functional layers. To quantitatively illustrate the ideality factor difference, the voltage-dependent carrier concentration of QLEDs was experimentally determined by combining TRC and TREL measurements. As described in our previous work^23^, the dynamics of electron and hole in the rising stage of TREL can be approximated as Eq. 14, while the time-dependent EL rising of TREL curves can be described as equation 15.

14 $$\frac{{dp}}{{dt}}=\frac{{dn}}{{dt}}=i-{r}_{1}{np}$$

15a $$L={\tanh }^{2}\left(K(t-{t}_{{\rm{d}}})\right)$$

15b $$K=\sqrt{{r}_{1}i}$$

Where *r*_1_, *n*, *p*, *t*_d_ and *i* represent Bimolecular Recombination Coefficient, electron concentration, hole concentration, delay time, and carrier injection rate ($i=\frac{I}{q}$), respectively.

**Fig. 3:**
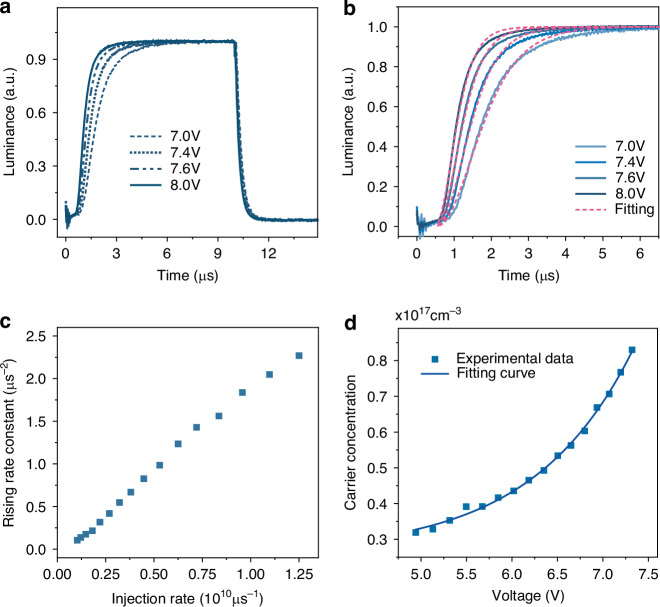
Transient electroluminescence (TREL) analysis of a quantum dot light-emitting diodes (QLED). **a** TREL curve of a typical QLED device. **b** Fitting of the rising edge in the TREL curve. **c** *K - i* plot derived from the rising edge fitting, where *K* represents the rising rate constant. **d** Carrier concentration versus voltage diagram calculated from the TREL fitting results and its fitting curve

Figure 3a, b show the fitted TREL curves of QLEDs with Eqs. 15a and 15b. The excellent fitting and the linear relationship between *K*^2^ and *i* validate the description of QLED operation with Eq. 14 and 15 (Fig. 3c). According to Eq. 14, a steadily operated QLED device typically has a constant electron and hole concentration, thus $\frac{{dp}}{{dt}}=\frac{{dn}}{{dt}}=0$, therefore $n=p=\sqrt{\frac{i}{{r}_{1}}}=\frac{i}{K}$. The carrier injection rate under different voltages can be calculated from transient current (TRC) curves, while *K* can be extracted by fitting the TREL curves of QLEDs. Therefore, the carrier concentration at different voltages can be calculated. As demonstrated in Fig. 3d, a typical QLED device exhibits a voltage-dependent carrier concentration profile with an exponential increase of $n={n}_{0}{e}^{\frac{V}{m{\phi }}}+C$, where *m* ≈ 42. Fig. S2 summarizes the JVL, EQE, TRC, and TREL curves of this typical device. These observed exponential growth characteristics are consistent with the theoretical framework of conventional semiconductor PN junctions. However, the coefficient in the exponential term deviates significantly from the silicon-based devices, which aligns with the explanation of ideality factor deviations proposed in our model.

To further analyze the difference of ideality factors, correlations were performed between the I–V curves of the QLED device and the corresponding sub-device with different function layers. Seventy-nine sub-devices and thirty QLED devices were fabricated, which are grouped into four categories. Fig. S14 summarizes the average values and 95% confidence intervals of key fitting parameters for the four QLED device groups and their corresponding sub-devices. The original data are described in Figs. S4–S8 and Tables S2–S9. Considering the influence of series resistance (*R*_s_), Eq. 16 was adapted for fitting I–V curves.

16 $$I={I}_{0}\left({e}^{\frac{q\left(V-I{R}_{{\rm{s}}}\right)}{mkT}}-1\right)$$

Specially, the transport properties of some modified ZMO and TFB do not apply to the hopping model, the sub-devices of these materials show linear I–V curves (Figs. S4b, S5d). In this case, the voltage distribution of these layers can be treated as an additional series resistance, which has been considered with Eq. 16 (see SI). Here *m*_HTL_, *m*_QD_ and *m*_ETL_ represent the characteristic factors of the hole transport layer (HTL), quantum dot (QD) layer, and electron transport layer (ETL), respectively. m* is the ideality factor predicted from the I–V curves of sub-devices using Eq. 13. *m* is the ideality factor fitted from I–V curves of QLED devices. Both of m* and *m* can describe the characteristics of QLED. The value of *p* is defined as *n*/*m* to compare theoretical predictions and empirical results.

Based on the results in the Fig. S14, the *p*-values of device group i-iii are 1.027, 1.003, 0.867, respectively and the *p*-values of device group iv is 0.673. The deviation in device group iv arises from a recombination reaction order below the ideal value of 2, suggesting the enhanced effects of non-radiative recombination. The details are discussed in the error analysis section. The consistency of m and m* supports the proposed explanation of large ideality factor in QLEDs. These results also confirm that Eq. 13 can precisely describe the relationship between the ideality factors of a QLED device and the characteristic factors of function layers. Based on the above results, the voltage divisions of functional layers in a typical QLED device vary the values of *V*_f_, thus affecting the carrier concentration of each layer. Therefore, the voltage-dependent carrier concentration accounts for the deviation from ideality factors.

**Fig. 4:**
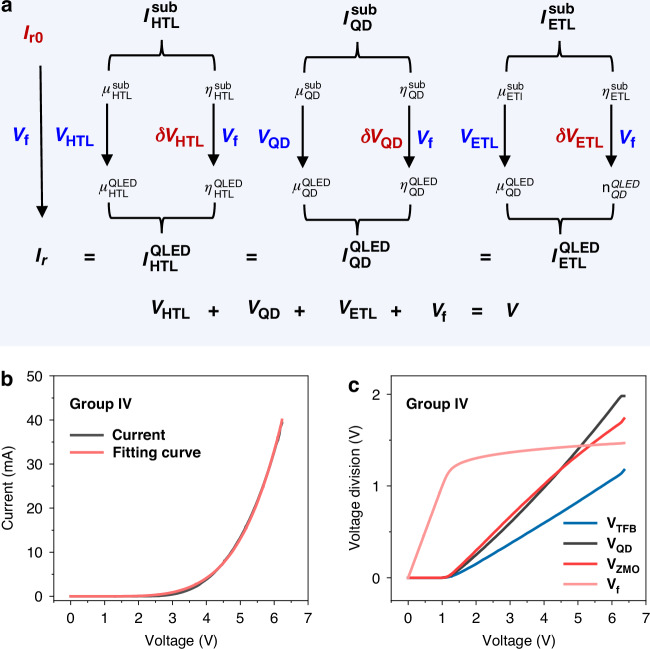
Numerical simulation and experimental validation of QLED physical parameters. **a** Schematic diagram of the relationships among physical parameters. **b** Comparison between the fitted I–V curve (simulation) and the original experimental I–V characteristics of the QLED (group 4). **c** Simulated voltage distribution across functional layers of the QLED as a function of applied voltage

Figure 4a summarizes the correlations between key physical parameters of functional layers, including mobility, carrier concentration, voltage divisions, current and total voltage. Based on Eq. 3, the parameters of *δV*_HTL_, *δV*_QD_ and *δV*_ETL_ associated with the equilibrium carrier concentrations of functional layers in QLEDs under zero voltage with the sub-devices. Based on Eqs. 7 and 8, *V*_f_ varies the carrier concentrations of functional layers, while *V*_HTL_, *V*_QD_ and *V*_ETL_ vary the carrier mobility of each correlation functional layer under applied voltage. Additionally, *V*_f_ also determines the relationship between the intrinsic recombination current (*I*_r0_) and its operational recombination current *I*_r_ based on Eq. 9. These interdependencies establish a quantitative link between the I–V behavior of sub-devices and the corresponding functional layers in QLEDs. Due to the complexity in mathematically describing charge transport within the functional layers of these sub-devices, a numerical simulation method was developed to reconstruct the I–V characteristics of QLED devices through integration of sub-device experimental data with key physical parameters (*δV*_HTL_, *δV*_QD_, *δV*_ETL_ and *I*_r0_).

By fitting the I–V curves of sub-devices and corresponding QLED through the aforementioned numerical simulation methods, key physical parameters can be extracted, and the relationships between the applied voltage and voltage distribution of each functional layer can be established. Figure 4b demonstrates the fitting performance for Group iv devices, while Fig. 4c maps the simulated voltage partitioning across functional layers against applied voltage. Based on the fitting results, the voltage drop across the nanocrystal-based PN junction can be derived. Before the turn-on voltage, the applied voltage primarily drops on the potential barrier region to modulate carrier concentration. As the voltage increases, a significant voltage drop was observed at the functional layer. This result aligns consistently with previous physical understanding. The extracted parameters include *δV*_HTL_ = 0.65 V, *δV*_QD_ = 0.72 V, *δV*_ETL_ = 0.64 V, and *I*_r0_ = 1.019 × 10^−26^ A. Specifically, the intrinsic carrier concentration of the QD material can be further calculated by combining the equation $q{r}_{1}{V}_{{\rm{m}}}{n}_{{\rm{i}}}^{2}={I}_{{\rm{r}}0}$ with the bimolecular recombination coefficient (*r*_1_) derived from TREL measurements. Figs. S8 and S9 present the fitting results for all groups, with Fig. S10 displaying the fitting outcomes plotted on a logarithmic scale. Fig. S11 shows the voltage distribution of functional layers versus applied voltage for all device groups, as derived from numerical fitting. Additionally, Tables S10–S13 present the confidence intervals and correlation matrices of these fitting results. All numerically fitted parameters exhibit relatively narrow confidence intervals. However, strong correlations persist among the fitted parameters, indicating their non-independent nature. This may be an inherent limitation of the nanocrystal-based PN junction model.

## Discussion

By adapting the theory of silicon based PN junctions, the models of nanocrystal-based PN junctions in QLEDs are established, which can illustrate the correlations between the I–V curves of functional layer-based sub-devices and the corresponding QLED devices. Based on the measured I–V curves and the corresponding fitting, the ideality factor of a typical QLED device can be mathematically and experimentally correlated with the characteristic factor of corresponding sub-devices, which account for the deviation of the ideality factor. We further summarize the correlations between key physical parameters of functional layers in QLED, including mobility, carrier concentration, voltage divisions, current, and total voltage. Furthermore, numerical simulations were applied to extract key physical parameters and establish the relationships between the applied voltage and voltage distribution of each functional layer. Importantly, the model emphasizes the critical influence of carrier concentration on operation QLED. In addition, the model can be also adapted to describe other nanoscale semiconductor devices.

## Materials and methods

All the QLEDs fabricated in this work are bottom-emission-device with a construction of glass/ITO/HIL/HTL/QDs/ETL/cathode. Ag was used as cathode in Group Ⅰ and Al was used as the cathode in other groups. The materials and fabrication processes for all devices in Group Ⅰ were provided by TCL. For groups Ⅱ-Ⅳ, the hole injection layer (HIL) utilizes PEDOT:PSS material sourced from Xi’an Polymer Light Technology Corp, while the hole transport layer (HTL) employs TFB material supplied by Xi’an Polymer Light Technology Corp. Regarding the electron transport layer (ETL), both Groups II–III and Group Ⅳ use ZMO (ZnMgO) provided by TCL. The magnesium (Mg) doping concentrations of Group Ⅳ are higher than in Groups II–III. The quantum dot (QD) materials of Groups Ⅰ and Ⅳ are supplied by TCL. The quantum dots in Groups II and III devices were synthesized in-house, with the detailed fabrication procedure outlined below: Blue CdZnSe/ZnSe/ZnS QDs: 0.8 mmol ZnAc_2_ with 1 mL OA and 9 mL ODE in a 50 mL three-neck flask were degassed at 120 °C under vacuum for 1 h, followed by heating at 300 °C under argon. Then, 0.5 mL of 0.5 M Se-DPP precursor was swiftly injected into the flask and reacted for 30 min. For the ZnSe shell, 2 mL of 0.5 M Zn(OA)_2_ and 0.4 mL 2 M Se-TOP precursor were injected and reacted for 30 min. For the ZnS shell with different thickness (the ZnS shell thickness of group III is higher than that of Group II), 1 mL 0.5 M Zn (OA)_2_ and 0.1 or 0.2 mL 2 M S-TOP precursor were injected and reacted for 10 or 20 min. The solution was then cooled to room temperature, followed by purification with ethanol and hexane, and dispersed into octane for device fabrication. Due to commercial confidentiality agreements, detailed specifications of the TCL-provided material cannot be disclosed.

The devices of group II–IV were fabricated on ITO glass substrates with a sheet resistance of ~20 Ω sq^−1^. Before device fabrication, the substrates were sequentially cleaned with deionized water, acetone, and isopropanol for 15 min each, followed by a 15-min UV-ozone treatment in ambient air to enhance surface wettability. The spin-coating processes for each functional layer were performed as follows: PEDOT:PSS was spin-coated at 5500 rpm for 1 min, followed by annealing at 150 °C in ambient air for 15 min, after which the coated substrate was transferred into a nitrogen-filled glovebox. The TFB layer was spin-coated at 3000 rpm for 1 min and annealed at 150 °C for 20 min. The QDs were deposited via spin-coating at 4000 rpm for 1 min and thermally treated at 80 °C for 30 min. The ZMO solution was spin-coated at 4000 rpm for 1 min and annealed at 100 °C for 20 min. The spin concentration of TFB is 8 mg mL^−1^. The spin concentration of QDs is 20 mg mL^−1^. The spin concentration of the ZMO solution is 20 mg mL^−1^. These multilayer samples were then loaded into a custom high vacuum deposition chamber (background pressure, ~3 × 10^−7^ torr) to deposit the top Al cathode (80 nm thick) patterned by an in-situ shadow mask to form an active device area of 4 mm^2^. Each device included two substrates with eight test points, and the averaged fitted data from these points were adopted as representative values.

Current-luminance-voltage characteristics were measured using an Agilent 4155C semiconductor parameter analyzer with a calibrated Newport silicon diode. The luminance was calibrated using a Minolta luminance meter (LS-100) according to the suggested method. The electroluminescence spectra were obtained with a JASCO FP750 spectrometer and a Keithley 2400 power source. As described in our previous work^23^, TREL and TRC measuring equipment are composed of an Oscilloscope, a Detector, a Voltage Pulse Generator, a rheostat, and several wires. The model of Oscilloscope, Detector and Voltage Pulse Generator is DPO 7104, HAMATSO 012702-11 and SDG 5162, respectively.

As described before, the equivalent circuit of actual device structures incorporates a linear resistance in series with the diode. Thus, the direct application of the Shockley equation to fit I–V curves of the aforementioned devices would introduce significant errors due to the unaccounted series resistance (*R*_s_). We employ two approaches to address this challenge. The first approach involves direct fitting of the implicit function described by Eq. 16, and we developed a python-based code to fit the parameters of this implicit equation. The second approach leverages our previously established transient current methodology to extract the series resistance^23^. With this value, we replace the applied voltage *V* with the corrected junction voltage ${V}^{{\prime} }=V-I* R{\rm{s}}$, thereby enabling accurate parameter extraction through the conventional Shockley equation. Furthermore, since the primary source of *R*s originates from the electrode resistance, the linear resistance of devices with an ITO/Al structure can also be used to approximate the value of Rs. Fig. S12 demonstrates the influence of series resistance (*Rs*) on parameter extraction.

## Supplementary information

Supplemental Material

## Data Availability

The data that support the findings of this work are available from the corresponding author upon reasonable request. All data requests will be handled by H.Z., who can be contacted at hzzhong@bit.edu.cn.
