# Supplementary material for: A nanocrystal-based PN junction model for quantum dot light-emitting diodes
Source: Light Sci Appl. 2026 Jul 17;15:322. doi: 10.1038/s41377-026-02356-9 (PMC13379575; doi:10.1038/s41377-026-02356-9)
Supplement: Supplementary file 1 — Supplemental Material [file 41377_2026_2356_MOESM1_ESM.pdf]

Supplementary Information for:

A nanocrystal-based PN junction model for quantum dots light-emitting diodes

Hui Bao[1], Seyed Mehdi Sattari-Esfahlan[1], Haizheng Zhong[1]*

[1]MIIT Key Laboratory for Low-Dimensional Quantum Structure and Devices, School of Materials Science & Engineering, Beijing Institute of Technology, Beijing 100081, China.

*Corresponding Author's E-mail: hzzhong@bit.edu.cn

Part 1. Derivation process

First, the IV characteristics of sub-devices (HTL, QD, and ETL) are expressed as:

$$I_{\mathrm{HTL}} = p_{\mathrm{HTL}} q S A_{\mathrm{HTL}}\, \mathrm{e}^{\frac{V}{m_{\mathrm{HTL}}\Phi}} \quad \text{S1}$$

$$I_{\mathrm{QD}} = n_{\mathrm{QD}} q S A_{\mathrm{QD}}\, \mathrm{e}^{\frac{V}{m_{\mathrm{QD}}\Phi}} \quad \text{S2}$$

$$I_{\mathrm{ETL}} = n_{\mathrm{ETL}} q S A_{\mathrm{ETL}}\, \mathrm{e}^{\frac{V}{m_{\mathrm{ETL}}\Phi}} \quad \text{S3}$$

Where p_{HTL}, n_{QD}, and n_{ETL} represent carrier concentration of HTL, QD, and ETL, respectively. A_{HTL} and m_{HTL} are empirical parameters related to material-specific hopping processes in HTL layers. A_{QD} and m_{QD} are empirical parameters related to material-specific hopping processes in QD layers. A_{ETL} and m_{ETL} are empirical parameters related to material-specific hopping processes in ETL layers.

In operated QLED devices, under the combined effects of the depletion layer formation process and the influence of an external electric field on the depletion layer, the transport current of function layers can be expressed as:

$$I_{\mathrm{HTL}} = p_{\mathrm{HTL}} q S A_{\mathrm{HTL}}\, \mathrm{e}^{\frac{V_{\mathrm{HTL}}}{m_{\mathrm{HTL}}\Phi} - \frac{\delta V_{\mathrm{HTL}}}{\Phi}} = p_{\mathrm{HTL0}} q S A_{\mathrm{HTL}} \mathrm{e}^{\frac{V_{\mathrm{HTL}}}{m_{\mathrm{HTL}}\Phi} + \frac{V_{\mathrm{f}}}{2\Phi} - \frac{\delta V_{\mathrm{HTL}}}{\Phi}} \quad \text{S4}$$

$$I_{\mathrm{QD}} = n_{\mathrm{QD}} q S A_{\mathrm{QD}}\, \mathrm{e}^{\frac{V_{\mathrm{QD}}}{m_{\mathrm{QD}}\Phi} - \frac{\delta V_{\mathrm{QD}}}{\Phi}} = n_{\mathrm{QD0}} q S A_{\mathrm{QD}} \mathrm{e}^{\frac{V_{\mathrm{QD}}}{m_{\mathrm{QD}}\Phi} + \frac{V_{\mathrm{f}}}{2\Phi} - \frac{\delta V_{\mathrm{QD}}}{\Phi}} \quad \text{S5}$$

$$I_{\mathrm{ETL}} = n_{\mathrm{ETL}} q S A_{\mathrm{ETL}}\, \mathrm{e}^{\frac{V_{\mathrm{ETL}}}{m_{\mathrm{ETL}}\Phi} - \frac{\delta V_{\mathrm{ETL}}}{\Phi}} = n_{\mathrm{ETL0}} q S A_{\mathrm{ETL}} \mathrm{e}^{\frac{V_{\mathrm{ETL}}}{m_{\mathrm{ETL}}\Phi} + \frac{V_{\mathrm{f}}}{2\Phi} - \frac{\delta V_{\mathrm{ETL}}}{\Phi}} \quad \text{S6}$$

Where δV_{HTL}, δV_{QD} and δV_{ETL} characterize the influence of depletion layer formation on the respective functional layers. V_{HTL}, V_{QD} and V_{ETL} characterize the voltage drop on each functional layer. V_{f} represent the quasi-fermi level splitting voltage of depletion layers under external electric filed. For simplified calculation, we set $p_{\mathrm{HTL0}} q S A_{\mathrm{HTL}} \mathrm{e}^{-\frac{\delta V_{\mathrm{HTL}}}{\Phi}} = B_{\mathrm{HTL}}$, $n_{\mathrm{QD0}} q S A_{\mathrm{QD}} \mathrm{e}^{-\frac{\delta V_{\mathrm{QD}}}{\Phi}} = B_{\mathrm{QD}}$, $n_{\mathrm{ETL0}} q S A_{\mathrm{ETL}} \mathrm{e}^{-\frac{\delta V_{\mathrm{ETL}}}{\Phi}} = B_{\mathrm{ETL}}$.

Based on the time-resolved electroluminescence (TREL) result, we assume that bimolecular recombination dominates in the QD layer. Thus I_r can be express as equation S7:

$$I_{\mathrm{r}} = q r_1 n_{\mathrm{QD}} p_{\mathrm{QD}} V_m = q r_1 V_m n_{\mathrm{i}}^2 e^{\frac{V_{\mathrm{f}}}{\Phi}} \quad \text{S7}$$

Similarly, for simplify calculation, we set $qr_1V_m n_{\mathrm{i}}^2 = I_{\mathrm{r0}}$。

The relationship of these current and voltage will follow equation S8 and equation S9:

$$I = I_{\mathrm{r}} = I_{\mathrm{HTL}} = I_{\mathrm{ETL}} = I_{\mathrm{QD}} \tag{S8}$$

$$V_1 + V_2 + V_3 + V_{\mathrm{f}} = V \tag{S9}$$

According to $I_r = I_{HTL}$, we can get equation S10.

$$\phi ln\frac{I_{\mathrm{r0}}}{B_{\mathrm{HTL}}} + \frac{V_{\mathrm{f}}}{2} = \frac{V_{\mathrm{HTL}}}{m_{\mathrm{HTL}}} \tag{S10}$$

According to $I_r = I_{QD}$, we can get equation S11:

$$\phi ln\frac{I_{\mathrm{r0}}}{B_{\mathrm{QD}}} + \frac{V_{\mathrm{f}}}{2} = \frac{V_{\mathrm{HTL}}}{m_{\mathrm{QD}}} \tag{S11}$$

According to $I_r = I_{ETL}$, we can get equation S12:

$$\phi ln\frac{I_{\mathrm{r0}}}{B_{\mathrm{ETL}}} + \frac{V_{\mathrm{f}}}{2} = \frac{V_{\mathrm{HTL}}}{m_{\mathrm{ETL}}} \tag{S12}$$

According to equation S10,S11,S12 and S9, we can get the relationships of current and applied voltage:

$$I = I_r = qr_1V_m n_i^2 e^{\frac{qV_f}{kT}} = B_r e^{\frac{V-A}{m\phi}} = I_s e^{\frac{V}{m\phi}} \tag{S13}$$

here

$$A = \phi\left(m_{\mathrm{HTL}} ln\frac{I_{\mathrm{r0}}}{B_{\mathrm{HTL}}} + m_{\mathrm{QD}} ln\frac{I_{\mathrm{r0}}}{B_{QD}} + m_{ETL} ln\frac{I_{\mathrm{r0}}}{B_{\mathrm{ETL}}}\right) \tag{S14}$$

$$I_s = I_{\mathrm{r0}} e^{\frac{-A}{m\phi}} \tag{S15}$$

$$m = \frac{2 + m_{\mathrm{ETL}} + m_{\mathrm{QD}} + m_{\mathrm{HTL}}}{2} \tag{S16}$$

In our model, we assume that bimolecular recombination dominates in the QD layer. However, due to the potential presence of defect-mediated or interface-induced first-order non-radiative recombination in practical quantum dot layers, bimolecular recombination cannot precisely characterize low-efficiency QLED devices. To address this, we assume the average recombination order of the quantum dot layer to be l, the recombination current can be expressed as equation S17.

$$I_r = qr_2 n^l V_m = qr_2 V_m n_i^l e^{\frac{lV_{\mathrm{f}}}{2\phi}} = I'_{r0} e^{\frac{lV_{\mathrm{f}}}{2\phi}} \tag{S17}$$

Where r_2 represent the average recombination rate. Apply equation S17 to replace

equation S13, we can get:

$$I = I_r = I'_{r0} e^{\frac{lV_f}{2\Phi}} = I'_{r0} e^{\frac{V-A}{m'\Phi}} = I_s{}' e^{\frac{V}{m'\Phi}} \qquad \text{S18}$$

Here we have

$$I_s{}' = I'_{r0} e^{\frac{-A}{m'\Phi}} \qquad \text{S19}$$

$$m' = \frac{l-1}{l}\left(m_{\mathrm{ETL}} + m_{\mathrm{QD}} + m_{\mathrm{HTL}}\right) + \frac{2}{l} \qquad \text{S20}$$

As derived from Equation S20, the ideality factor exhibits values lower than those pre dicted by Equation S16 when the recombination order falls below 2.

Furthermore, our previous derivation assumed symmetrical splitting of the electron and hole quasi-Fermi levels under the hypothesis of identical transport rates in the quantum dot (QD) layer. However, as this assumption may not fully align with practical conditions, we propose that the electron and hole transport currents (I_{QDn}, I_{QDp}) in the QD layer can be modeled by the following expressions:

$$I_{\mathrm{QDn}} = n_{\mathrm{QD}} q S A_{\mathrm{QDn}}\, \mathrm{e}^{\frac{V_{\mathrm{QD}}}{m_{\mathrm{QDn}}\Phi} - \frac{\delta V_{\mathrm{QD}}}{\Phi}} = n_{\mathrm{QD0}} q S A_{\mathrm{QDn}}\, \mathrm{e}^{\frac{V_{\mathrm{QD}}}{m_{\mathrm{QDn}}\Phi} + \frac{E_{\mathrm{fn}} - E_{\mathrm{f}}}{q\Phi} - \frac{\delta V_{\mathrm{QD}}}{\Phi}} \qquad \text{S21}$$

$$I_{\mathrm{QDp}} = p_{\mathrm{QD}} q S A_{\mathrm{QDp}}\, \mathrm{e}^{\frac{V_{\mathrm{QD}}}{m_{\mathrm{QDp}}\Phi} - \frac{\delta V_{\mathrm{QD}}}{\Phi}} = p_{\mathrm{QD0}} q S A_{\mathrm{QDp}}\, \mathrm{e}^{\frac{V_{\mathrm{QD}}}{m_{\mathrm{QDp}}\Phi} + \frac{E_{\mathrm{f}} - E_{\mathrm{fp}}}{q\Phi} - \frac{\delta V_{\mathrm{QD}}}{\Phi}} \qquad \text{S22}$$

The assumption that leakage current is absent will lead to $I_{QDn} = I_{QDp}$. Thus, we can get:

$$I_{\mathrm{QDn}} = I_{\mathrm{QDp}} = I_{\mathrm{QD}} = p_{\mathrm{QD0}} q S A_{\mathrm{QDp}} \mathrm{e}^{\frac{V_{\mathrm{QD}}}{m''\Phi} + \frac{V_f}{2\Phi} - \frac{\delta V_{\mathrm{QD}}}{\Phi} + C} \qquad \text{S23}$$

And

$$C = \frac{1}{2} ln \frac{n_{\mathrm{QD0}} A_{\mathrm{QDn}}}{p_{\mathrm{QD0}} A_{\mathrm{QDp}}} \qquad \text{S24}$$

$$\frac{1}{m''} = \frac{1}{2}\left(\frac{1}{m_{\mathrm{QDn}}} + \frac{1}{m_{\mathrm{QDp}}}\right) \qquad \text{S25}$$

Consequently, the asymmetric splitting of quasi-Fermi levels, arising from the disparity in electron and hole mobilities, also impacts the extracted ideality factor.

Part 2. Numerical simulation result

As revealed by the parameter correlations in Fig. 4a, we developed a numerical

simulation code that can compute IV characteristics of a QLED device by combining sub-device IV characteristics and four key physical parameters (δV_{HTL}, δV_{QD}, δV_{ETL} and I_{r0}). This code further enables experimental IV curve fitting via Python's curve-fit module, with outputs containing both aforementioned four key physical parameters and characteristics of functional layer voltage distribution versus external applied voltage. The IV curve fitting accuracies for all four device groups are demonstrated in Fig. S9, while their corresponding functional layer voltage distribution versus external applied voltage are systematically presented in Fig. S10 and Fig. S10. The fitted parameters across the four device groups are as shown in Table S10.

Part 3. Error analysis section

As stated at the conclusion of the main text, the potential sources of errors may include the following factors.

i) Errors of Rs determination during data fitting.

As previously mentioned, the TRC method enables Rs determination. However, measurement inaccuracies cannot be eliminated, which will affect the fitting of the ideal factor. To visually demonstrate the impact of Rs measurement errors on the ideality factor, Fig. S10 compares ideality factor variations derived from Rs-dependent fitting of two representative sub-devices' I-V characteristics. As shown in Fig. S10a, device 2 exhibits a relatively low current density, and device 1 operates at higher current levels. According to Fig. S10bcd, the fitting parameters (ideality factor, saturation dark current, and correlation coefficient between fitted/actual characteristics) of low-current devices exhibit insensitivity to variations in series resistance (Rs), while those of high-current devices demonstrate strong sensitivity to Rs changes. Consequently, when the overall device current is substantial, the fitting results are more susceptible to significant deviations.

ii) the recombination of SRH and/or Auger processes.

As indicated by equation S23, devices dominated by Shockley-Read-Hall (SRH) recombination exhibit an average recombination order below 2, consequently leading

to a reduced ideality factor. Conversely, Auger recombination-dominated systems display a recombination order exceeding 2, thereby elevating the ideality factor. In scenarios where multiple recombination mechanisms coexist, the interplay between these processes introduces significant complexity and unpredictability in ideality factor evolution.

iii) asymmetric quasi-Fermi level splitting between electrons and holes.

As theoretically derived in Equations S24–S28, the asymmetric splitting of quasi-Fermi levels originates from imbalanced electron and hole mobilities, necessitating the substitution of the QD material's characteristic factor with the equivalent parameter defined by Equation 5. However, experimental validation of this substitution remains challenging, thus, more discussion cannot be carried out in this context.

iv) electron/hole leakage currents.

The presence of leakage current introduces two critical errors in the fitting process. Firstly, it elevates the measured total current magnitude, which amplifies the voltage drop across the series resistance (Rs) and consequently reduces the extracted ideality factor. Secondly, the indeterminate voltage dependence inherent to leakage current itself directly compromises fitting accuracy. These combined effects significantly undermine the reliability of parameter extraction outcomes in device characterization.

In summary, we have systematically cataloged all potential sources of model uncertainty. Despite these complexities, the central tenet of our model remains robust: the deviation of the ideality factor originates from voltage-dependent co-modulation of both carrier concentration and mobility. This mechanism stands in marked contrast to conventional silicon-based p-n junctions, where the ideality factor primarily arises from voltage-driven carrier concentration variations alone.

Part 4. Figures and tables

Figures S1 show the IV characteristics of sub-devices under light and reverse voltage, respectively. Figures S2 show the IVL, EQE, TREL and TRC test results of a typical QLED. Figure S3 show the comparison of experimental and fitted I-V characteristics

for three representative sub-devices using four models. Figs. S4-S7 show the I-V characteristics of sub-devices in four groups and Fig. S8 show the I-V characteristics of corresponding QLED devices. It is noticed that the devices of groups 2, 3 and 4 use the same hole transport layers and hole inject layers. The devices of groups 2 and 3 using same electron transport layers. Figs. S9, S10, and S11 show the result of the numerical simulation. Figure S12 summarizes the impact of series resistance (Rs) on the extraction of diode parameters from IV characteristics. Figure S13 illustrates the relationship between doping concentration and depletion layer width in standard Si PN junctions. Figure S14 compares the predicted and experimentally fitted ideality factors (m) for QLED sub-devices. Table S1 presents the fitting parameters of four current-voltage (I-V) models for representative samples extracted from three characteristic sub-devices, along with their corresponding mathematical forms. Tables S2-S5 show the fitting result of sub-devices in groups 1-4, and Tables S6-S9 show the fitting result of corresponding QLED devices. Table S10 summarizes the numerical fitting results with 95% confidence intervals for all analyzed parameters. Tables S11-S14 present the correlation matrices of numerically fitted parameters for each device group (four groups total).

The device categorization was primarily based on experimental convenience and material availability during the initial validation phase of our theoretical model. We emphasize that the core physical framework (Eqs. 1-13) is universally applicable to QLEDs regardless of specific layer materials, as demonstrated by the consistent fitting accuracy across all four groups.

The light source used in Figure S1 is a PE-4000 model operating at 405 nm; this wavelength was selected due to material absorption characteristics. Similar current-voltage (I-V) characteristics were exhibited across all tested optical power levels.

Table S1. Fitting parameters of four IV models for representative samples from three characteristic sub-devices. mathematical forms:

$$1)\ I = I_0 e^{\frac{V-IRs}{M}},\ 2)\ I = I_0 e^{\sqrt{\frac{V-IRs}{M}}},\ 3)\ I = I_0 e^{(\frac{V-IRs}{M})^n},\ \ 4)\ I = I_0 (V-IRs)^n.$$

Device	form	I_0_CI	Rs_CI	M_CI	n_CI	R^2
ITO/ET	form1	6.60e-05±4.38e-	82.6±2.3	5.89e-01±1.18e-		0.9999

L/Al		06		02		
	form2	3.83e-06±4.08e-07	54.8±2.8	5.23e-02±1.83e-03		1.0000
	form3	2.41e-05±6.22e-06	69.3±2.9	2.69e-01±5.64e-02	0.74±0.05	1.0000
	form4	2.30e-04±1.81e-05	0.1±13.3		2.84±0.13	0.9997
ITO/HTL/Al	form1	2.47e-04±2.69e-05	44.1±2.9	6.79e-01±2.81e-02		0.9997
	form2	2.75e-05±2.62e-06	21.8±2.1	7.89e-02±3.18e-03		0.9999
	form3	4.59e-05±2.81e-05	25.5±5.1	1.38e-01±8.84e-02	0.57±0.09	0.9999
	form4	6.51e-04±6.31e-05	0.0±14.6		2.53±0.20	0.9992
ITO/QD/Al	form1	8.54e-04±1.63e-04	69.8±1.3	2.26e-01±2.23e-02		0.9998
	form2	2.17e-04±2.41e-05	62.7±0.8	4.22e-02±2.94e-03		1.0000
	form3	8.30e-05±5.22e-05	59.5±1.8	1.00e-02±9.76e-03	0.38±0.06	1.0000
	form4	1.00e-03±1.82e-02	58.3±10.4		100±18	0.9441

Table S2. fitting result of sub-device in group I.

Structure	Number	I_s	m*Φ	R	R_s
ITO/QD/Al	1	5.00E-06±2.84E-06	0.77±0.10	0.9991	315
	2	2.21E-05±9.14E-06	0.93±0.09	0.9996	315
	3	3.10E-06±1.80E-06	0.75±0.09	0.9991	315
	4	1.25E-05±5.63E-06	0.82±0.08	0.9995	315
	5	1.66E-06±7.87E-07	0.85±0.08	0.9995	320
	6	6.54E-07±3.04E-07	0.86±0.08	0.9994	320
	7	2.51E-05±6.45E-06	1.04±0.07	0.9998	320
ITO/ZMO/Al	1	1.42E-07±7.72E-08	0.47±0.03	0.9998	166
	2	1.69E-07±8.33E-08	0.47±0.03	0.9998	166
	3	1.57E-07±9.45E-08	0.47±0.03	0.9998	166
	4	1.22E-07±6.80E-08	0.46±0.03	0.9998	166
	5	1.89E-06±1.20E-06	0.58±0.05	0.9997	145
	6	3.54E-07±2.41E-07	0.50±0.04	0.9997	145
	7	4.90E-07±2.76E-07	0.50±0.03	0.9998	145

Table S3. fitting result of sub-device in group II.

Structure	Number	I_s	m*Φ	*R*	R_s
ITO/PEDOT:PSS/QD /Al	1	9.01E-04±1.13E-04	1.15±0.10	1.0000	888±109
	2	1.77E-03±1.83E-04	1.23±0.09	0.9997	544±60
	3	1.00E-02±9.91E-04	0.68±0.04	1.0000	97±3
	4	9.12E-03±2.55E-04	0.71±0.01	1.0000	80±1
	5	1.00E-02±5.46E-04	0.85±0.03	0.9999	98±3
	6	9.49E-03±4.19E-04	1.02±0.03	1.0000	151±4
	7	2.86E-03±1.29E-04	1.09±0.03	0.9999	220±10
	8	2.99E-03±1.91E-04	0.85±0.03	0.9999	172±6
ITO/PEDOT:PSS/TF B/Al	1	2.84E-03±1.77E-04	0.79±0.02	0.9999	31±2
	2	2.80E-03±9.95E-05	0.69±0.01	1.0000	39±1
	3	4.32E-03±1.05E-04	0.82±0.01	1.0000	35±1
	4	6.41E-03±2.13E-04	0.95±0.02	1.0000	48±1
	5	5.40E-03±1.65E-04	0.92±0.01	1.0000	60±1
	6	5.41E-03±3.65E-04	1.19±0.05	0.9999	86±8
	7	1.34E-03±6.06E-05	0.38±0.01	0.9999	319±2
	8	4.17E-03±3.75E-04	0.90±0.05	0.9999	222±11

Table S4. fitting result of sub-device in group III.

Structure	Number	Is	m*Φ	R	Rs
ITO/PEDOT:PSS/Q D/Al	1	1.41E-03±6.25E-05	1.55±0.05	0.9999	118±37
	2	2.96E-04±4.60E-05	0.90±0.07	0.9993	926±117
	3	1.94E-03±1.68E-04	1.41±0.09	0.9998	287±52
	4	2.35E-03±1.59E-04	1.49±0.07	0.9998	205±37
	5	1.38E-03±1.70E-04	1.32±0.11	0.9995	376±82
	6	7.45E-04±8.67E-05	1.09±0.08	0.9996	894±98
	7	1.72E-03±9.82E-05	1.45±0.05	0.9999	150±34
	8	1.66E-03±1.13E-04	1.44±0.07	0.9998	209±43

ITO/PEDOT:PSS/TFB/Al	1	2.84E-03±1.77E-04	0.79±0.02	0.9999	31±2
	2	2.80E-03±9.95E-05	0.69±0.01	1.0000	39±1
	3	4.32E-03±1.05E-04	0.82±0.01	1.0000	35±1
	4	6.41E-03±2.13E-04	0.95±0.02	1.0000	48±1
	5	5.40E-03±1.65E-04	0.92±0.01	1.0000	60±1
	6	5.41E-03±3.65E-04	1.19±0.05	0.9999	86±8
	7	1.34E-03±6.06E-05	0.38±0.01	0.9999	319±2
	8	4.17E-03±3.75E-04	0.90±0.05	0.9999	222±11

Table S5. fitting result of sub-device in group IV.

Structure	Number	I_s	$m*\Phi$	R	R_s
ITO/PEDOT:PSS/QD/Al	1	2.98E-02±1.24E-03	0.62±0.02	1.0000	75±1
	2	3.04E-02±1.04E-03	0.44±0.01	1.0000	74±0
	3	3.22E-02±1.03E-03	0.43±0.01	1.0000	63±0
	4	3.74E-02±2.08E-03	0.54±0.02	1.0000	66±1
	5	3.32E-02±2.48E-03	0.47±0.02	1.0000	71±1
	6	3.19E-02±1.10E-03	0.50±0.01	1.0000	67±0
	7	1.69E-02±2.80E-04	0.31±0.00	1.0000	83±0
ITO/PEDOT:PSS/TFB/Al	1	2.84E-03±1.77E-04	0.79±0.02	0.9999	31±2
	2	2.80E-03±9.95E-05	0.69±0.01	1.0000	39±1
	3	4.32E-03±1.05E-04	0.82±0.01	1.0000	35±1
	4	6.41E-03±2.13E-04	0.95±0.02	1.0000	48±1
	5	5.40E-03±1.65E-04	0.92±0.01	1.0000	60±1
	6	5.41E-03±3.65E-04	1.19±0.05	0.9999	86±8
	7	1.34E-03±6.06E-05	0.38±0.01	0.9999	319±2
	8	4.17E-03±3.75E-04	0.90±0.05	0.9999	222±11
ITO/PEDOT:PSS/ZMO/Al	1	2.96E-04±7.07E-06	0.55±0.00	1.0000	116±1
	2	4.15E-04±3.43E-06	0.55±0.00	1.0000	100±0
	3	5.08E-04±2.69E-05	0.64±0.01	0.9999	135±3

	4	1.28E-04±1.30E-05	0.42±0.01	1.0000	172±3
	5	3.43E-05±1.67E-06	0.39±0.00	1.0000	161±1
	6	9.05E-04±7.37E-06	0.64±0.00	1.0000	76±0
	7	2.36E-04±6.53E-06	0.59±0.00	1.0000	187±2
	8	7.25E-04±2.73E-05	0.56±0.01	1.0000	151±2

Table S6. fitting result of QLED in group I.

Number	Is	m*Φ	R	Rs
1	2.68E-06±1.58E-07	0.75±0.01	0.9998	547
2	3.27E-06±2.48E-07	0.77±0.01	0.9996	355
3	1.45E-06±6.16E-08	0.67±0.00	0.9999	336
4	5.57E-07±3.47E-08	0.59±0.00	0.9998	356
5	9.92E-07±3.73E-08	0.66±0.00	0.9999	319
6	3.09E-06±2.02E-07	0.78±0.01	0.9997	335
7	2.59E-06±1.73E-07	0.74±0.01	0.9997	321
8	7.20E-07±4.87E-08	0.62±0.00	0.9997	329

Table S7. fitting result of QLED in group II.

Number	Is	m*Φ	R	Rs
1	4.65E-06±3.88E-07	1.00±0.02	0.9998	822±45
2	1.16E-05±5.73E-07	1.06±0.01	0.9999	30±10
3	3.09E-06±1.80E-07	1.03±0.01	0.9999	202±35
4	8.78E-05±1.05E-05	0.81±0.02	0.9998	87±2
5	2.91E-05±3.18E-06	0.85±0.02	0.9998	166±5
6	8.84E-06±3.74E-07	0.98±0.01	0.9999	10±5
7	1.33E-09±1.67E-09	0.51±0.04	0.9635	155±506

Table S8. fitting result of QLED in group III.

Number	Is	m*Φ	R	Rs

1	5.61E-06±3.59E-07	0.91±0.01	0.9999	90±7
2	5.00E-05±3.42E-06	0.99±0.01	0.9999	109±4
3	6.64E-06±6.01E-07	1.03±0.02	0.9998	446±37
4	9.74E-05±1.56E-05	0.96±0.03	0.9995	104±6
5	4.74E-06±3.66E-07	1.02±0.01	0.9999	964±49
6	2.43E-05±5.20E-06	0.72±0.03	0.9996	289±8

Table S9. fitting result of QLED in Group IV.

Number	Is	m*Φ	R	Rs
**1**	**4.58E-05±5.71E-06**	**0.62±0.01**	**0.9999**	**80±1**
2	5.98E-05±1.08E-05	0.66±0.02	0.9998	73±1
3	3.65E-05±5.87E-06	0.80±0.02	0.9997	506±13
4	4.72E-05±9.26E-06	0.59±0.02	0.9999	75±1
5	4.75E-05±7.04E-06	0.59±0.01	0.9999	69±1
6	4.55E-05±7.30E-06	0.59±0.01	0.9999	74±1
7	1.41E-05±1.86E-06	0.50±0.01	1.0000	98±1

Table S10. Numerical fitting result with 95% cl.

	Group I	Group II	Group III	Group IV
Ir0(10^-29)	9205±1480	3434±812	417±53	1019±102
δVTFB	0.79±0.002	0.73±0.003	0.68±0.002	0.65±0.001
δVQD	0.59±0.002	0.72±0.003	0.62±0.002	0.72±0.001
δVZMO	0.59±0.002	0.78±0.003	0.71±0.003	0.64±0.001

Table S11. Parameter correlation matrix of numerical fitting results for Device Group I.

Correlation Matrix	I_{r0}(10^-29)	δV_{HTL}	δV_{QD}	δV_{ETL}
I_{r0}(10^-29)	1	-0.99791	-0.99693	-0.99542
δV_{HTL}	-0.99791	1	0.997395	0.989361

δV_{QD}	-0.99693	0.997395	1	0.985036
δV_{ETL}	-0.99542	0.989361	0.985036	1

Table S12. Parameter correlation matrix of numerical fitting results for Device Group II.

**Correlation Matrix**	$\boldsymbol{I_{\mathrm{r0}}}$**(10^-29)**	$\boldsymbol{\delta V_{\mathrm{HTL}}}$	$\boldsymbol{\delta V_{\mathrm{QD}}}$	$\boldsymbol{\delta V_{\mathrm{ETL}}}$
I_{r0} (10^-29)	1	-0.99987	-0.9998	-0.99494
δV_{HTL}	-0.99987	1	0.999502	0.994894
δV_{QD}	-0.9998	0.999502	1	0.993064
δV_{ETL}	-0.99494	0.994894	0.993064	1

Table S13. Parameter correlation matrix of numerical fitting results for Device Group III.

**Correlation Matrix**	**Ir0(10^-29)**	$\boldsymbol{\delta V_{\mathrm{HTL}}}$	$\boldsymbol{\delta V_{\mathrm{QD}}}$	$\boldsymbol{\delta V_{\mathrm{ETL}}}$
I_{r0} (10^-29)	1	-0.98632	-0.99616	-0.93197
δV_{HTL}	-0.98632	1	0.968959	0.891888
δV_{QD}	-0.99616	0.968959	1	0.932516
δV_{ETL}	-0.93197	0.891888	0.932516	1

Table S14. Parameter correlation matrix of numerical fitting results for Device Group IV.

**Correlation Matrix**	**Ir0(10^-29)**	$\boldsymbol{\delta V_{\mathrm{HTL}}}$	$\boldsymbol{\delta V_{\mathrm{QD}}}$	$\boldsymbol{\delta V_{\mathrm{ETL}}}$
I_{r0} (10^-29)	1	-0.98884	-0.99806	-0.99386
δV_{HTL}	-0.98884	1	0.980554	0.972529
δV_{QD}	-0.99806	0.980554	1	0.990966
δV_{ETL}	-0.99386	0.972529	0.990966	1

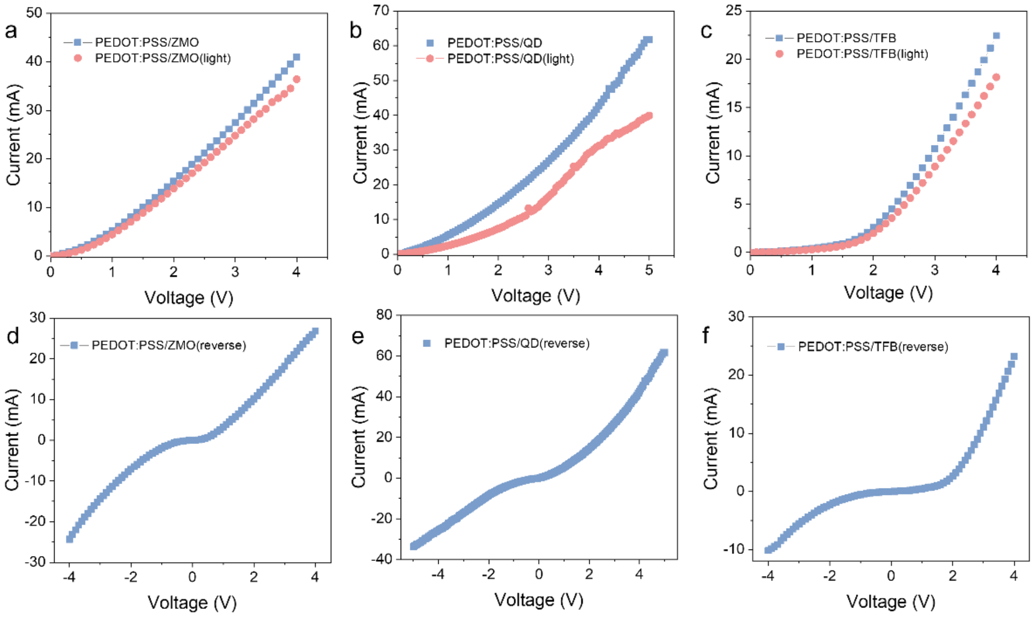

Figure S1. Current-voltage (I-V) characteristics of sub-devices under different operational conditions. **(a)-(c)** Comparative IV characteristics measured under illumination (red) and dark conditions (black) for **(a)** ITO/PEDOT:PSS/ZMO/Al; **(b)** ITO/PEDOT:PSS/QD/Al; (c) ITO/PEDOT:PSS/TFB/Al. (d-f). Corresponding reverse-bias IV characteristics for devices with **(d)** ITO/PEDOT:PSS/ZMO/Al; **(e)** ITO/PEDOT:PSS/QD/Al; **(f)** ITO/PEDOT:PSS/TFB/Al.

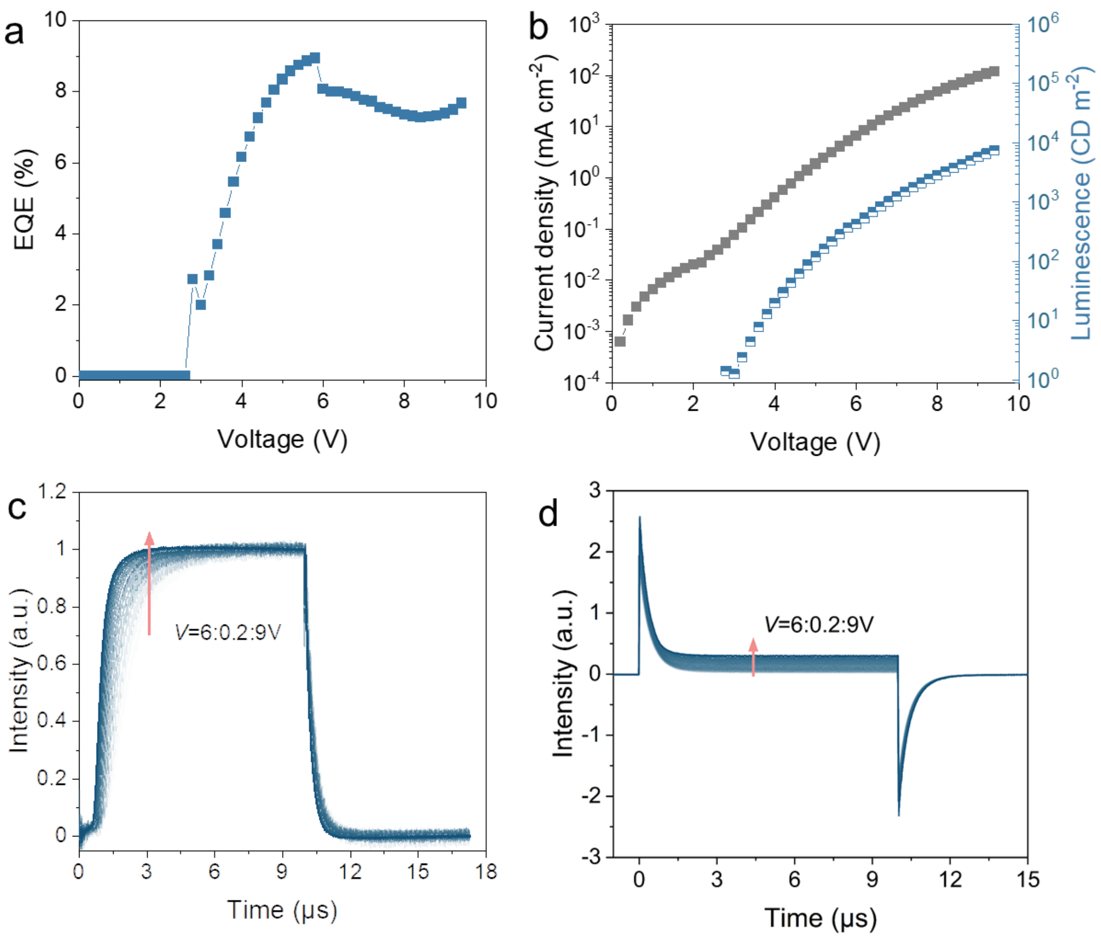

Figure S2. Device characterization and time-resolved optoelectronic measurements corresponding to the device configuration in Figure. 3. **(a)** External quantum efficiency (EQE) characteristics, **(b)** current density-voltage-luminance (J-V-L) characteristics, **(c)** time-resolved electroluminescence (TREL) characteristics, and **(d)** time-resolved current (TRC) characteristics.

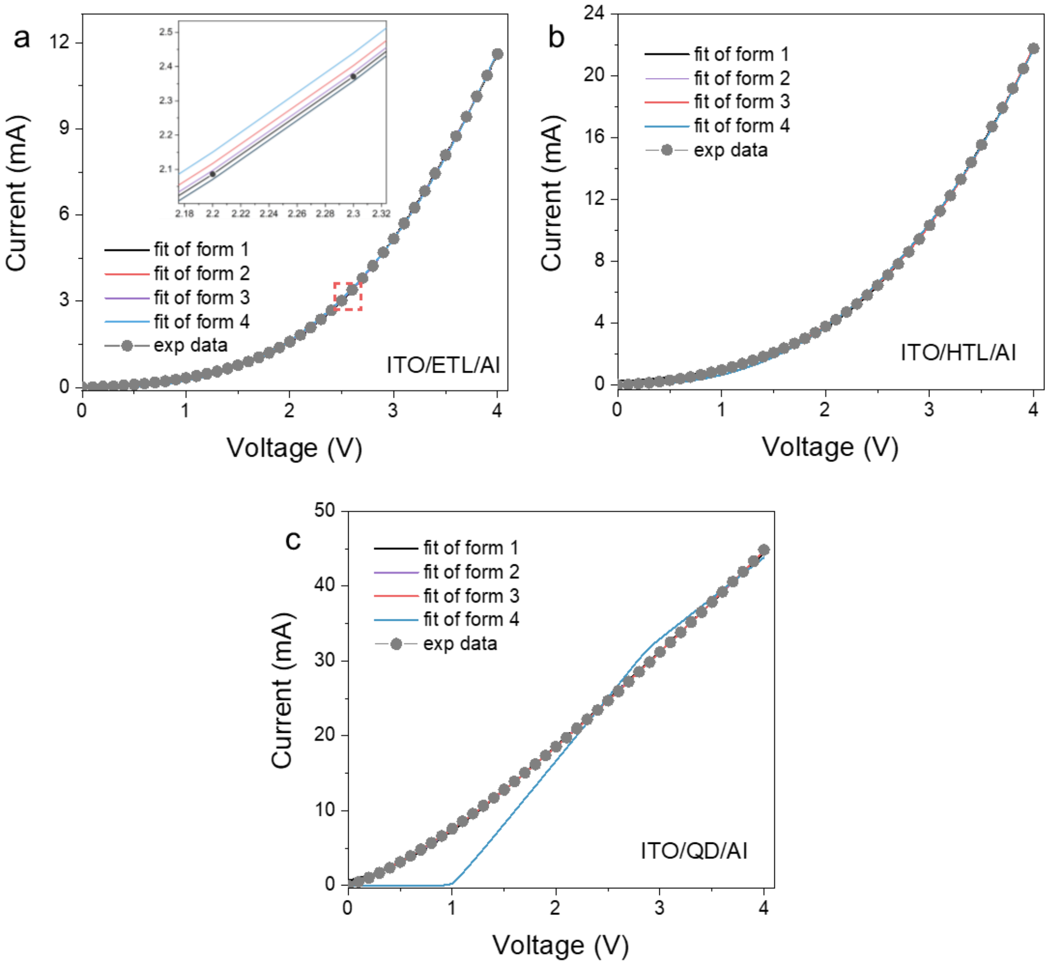

Figure S3. Comparison of experimental and fitted I-V curves for three representative sub-devices using four models. **(a)** ITO/ETL/Al, **(b)** ITO/HTL/Al, **(c)** ITO/QD/Al.

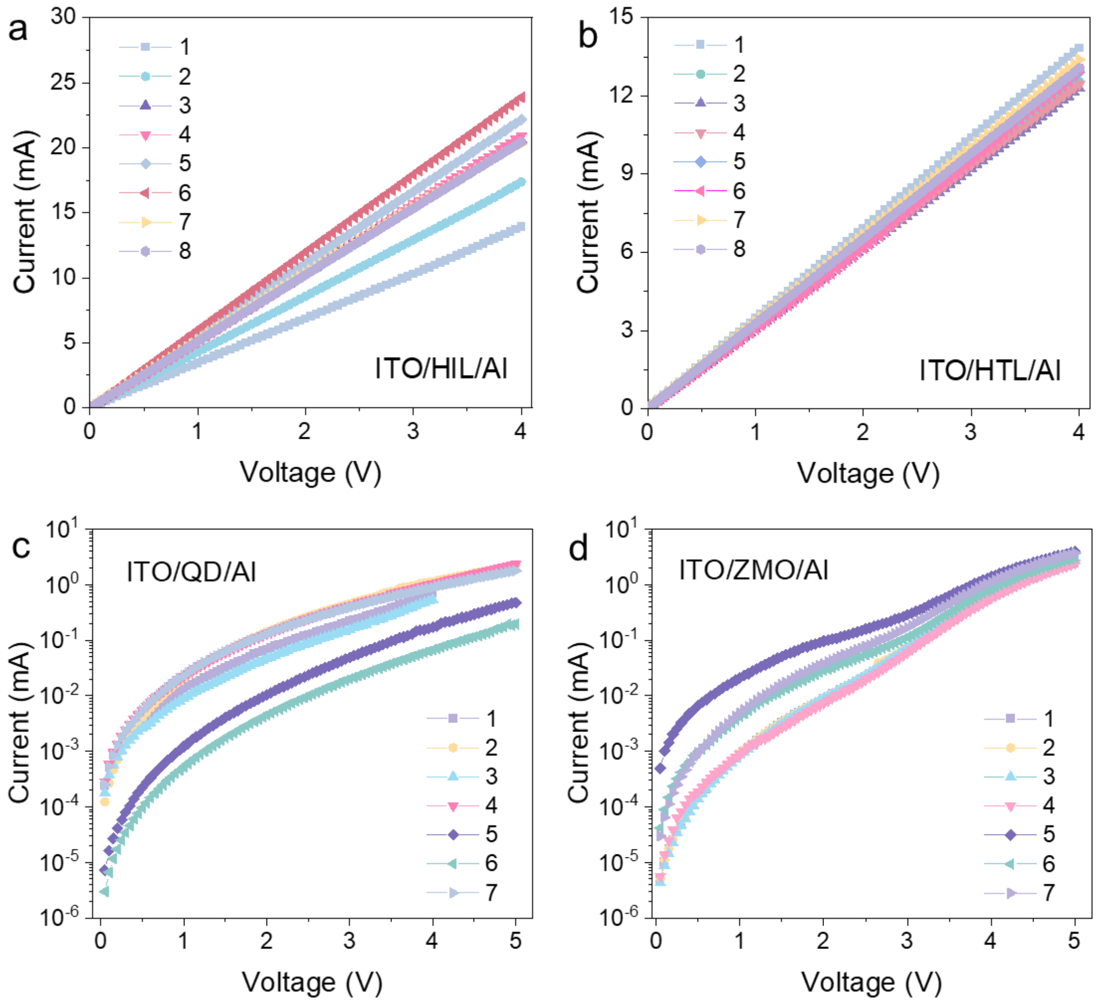

Figure S4. I-V characteristics of all sub-devices in group I: **(a)** ITO/PEDOT:PSS/Al structure; **(b)** ITO/TFB/Al structure; **(c)** ITO/QD/Al structure; **(d)** ITO/ZMO/Al structure.

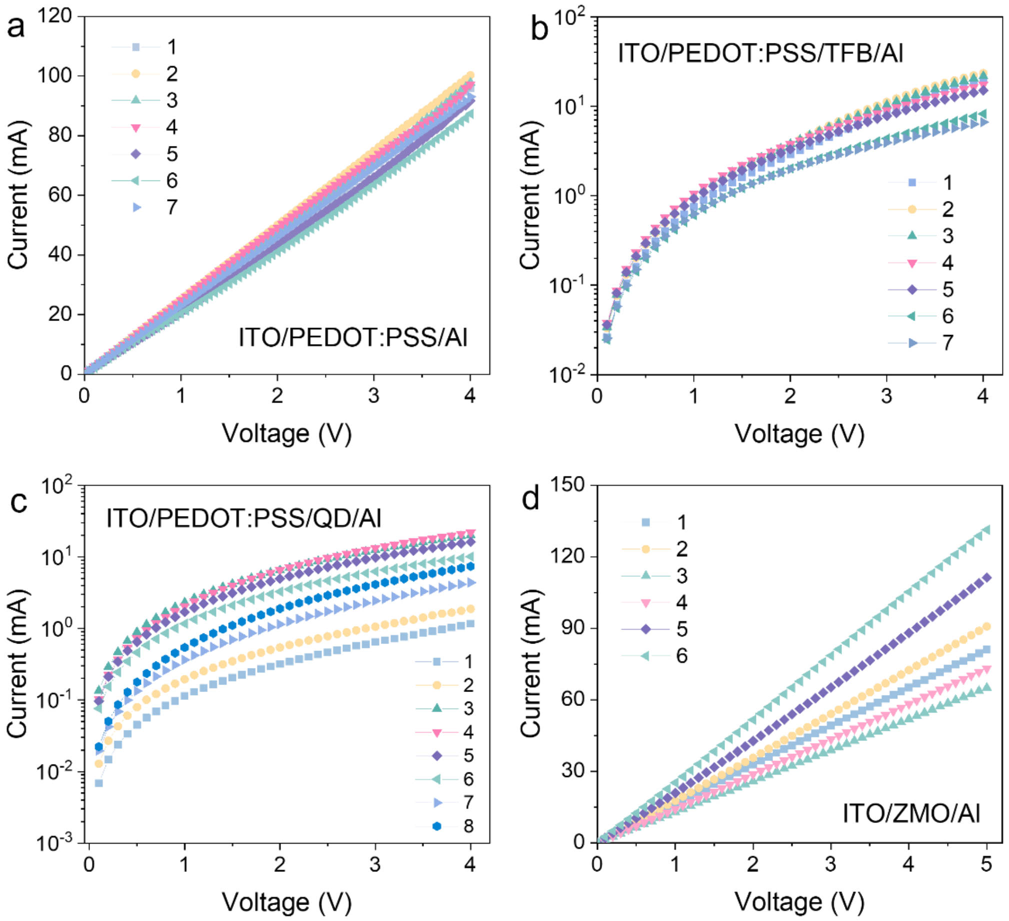

Figure S5. I-V characteristics of all sub-devices in group II: **(a)** ITO/PEDOT:PSS/Al structure; **(b)** ITO/PEDOT:PSS/TFB/Al structure; **(c)** ITO/PEDOT:PSS/QD/Al structure; **(d)** ITO/ZMO/Al structure.

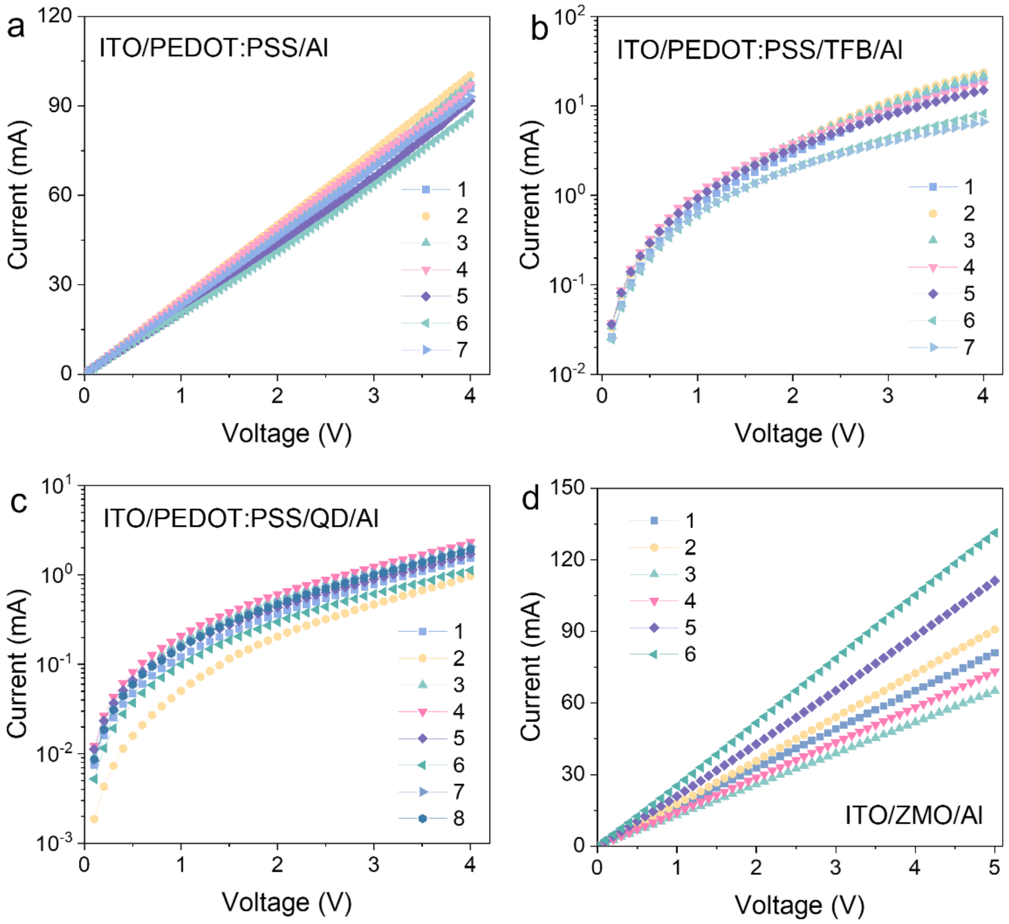

Figure S6. I-V characteristics of all sub-devices in group III: **(a)** ITO/PEDOT:PSS/Al structure; **(b)** ITO/PEDOT:PSS/TFB/Al structure; **(c)** ITO/PEDOT:PSS/QD/Al structure; **(d)** ITO/ZMO/Al structure.

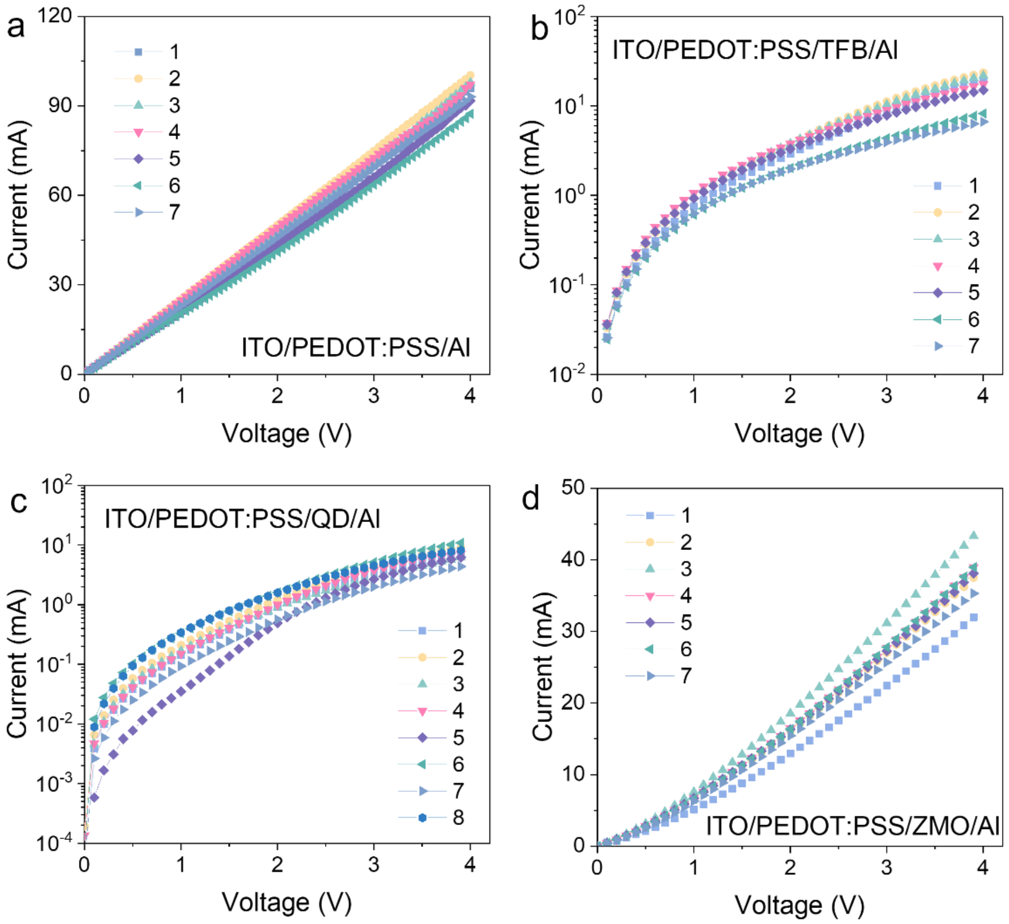

Figure S7. I-V characteristics of all sub-devices in Group IV: (a) ITO/PEDOT:PSS/Al structure; **(b)** ITO/PEDOT:PSS/TFB/Al structure; **(c)** ITO/PEDOT:PSS/QD/Al structure; **(d)** ITO/PEDOT:PSS/ZMO/Al structure.

.

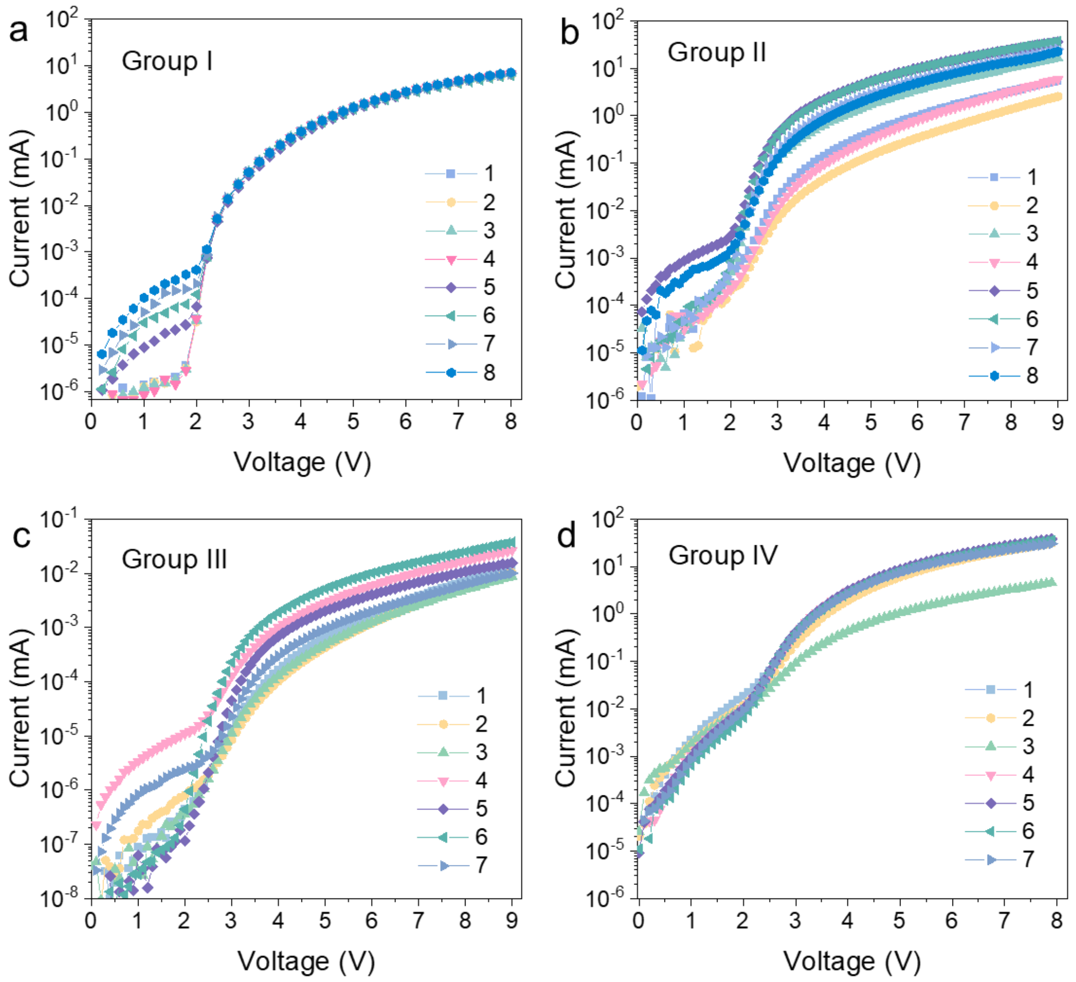

Figure S8. I-V characteristics of all QLED devices. **(a)** Group I, **(b)** Group II, **(c)** Group III, **(d)** Group IV.

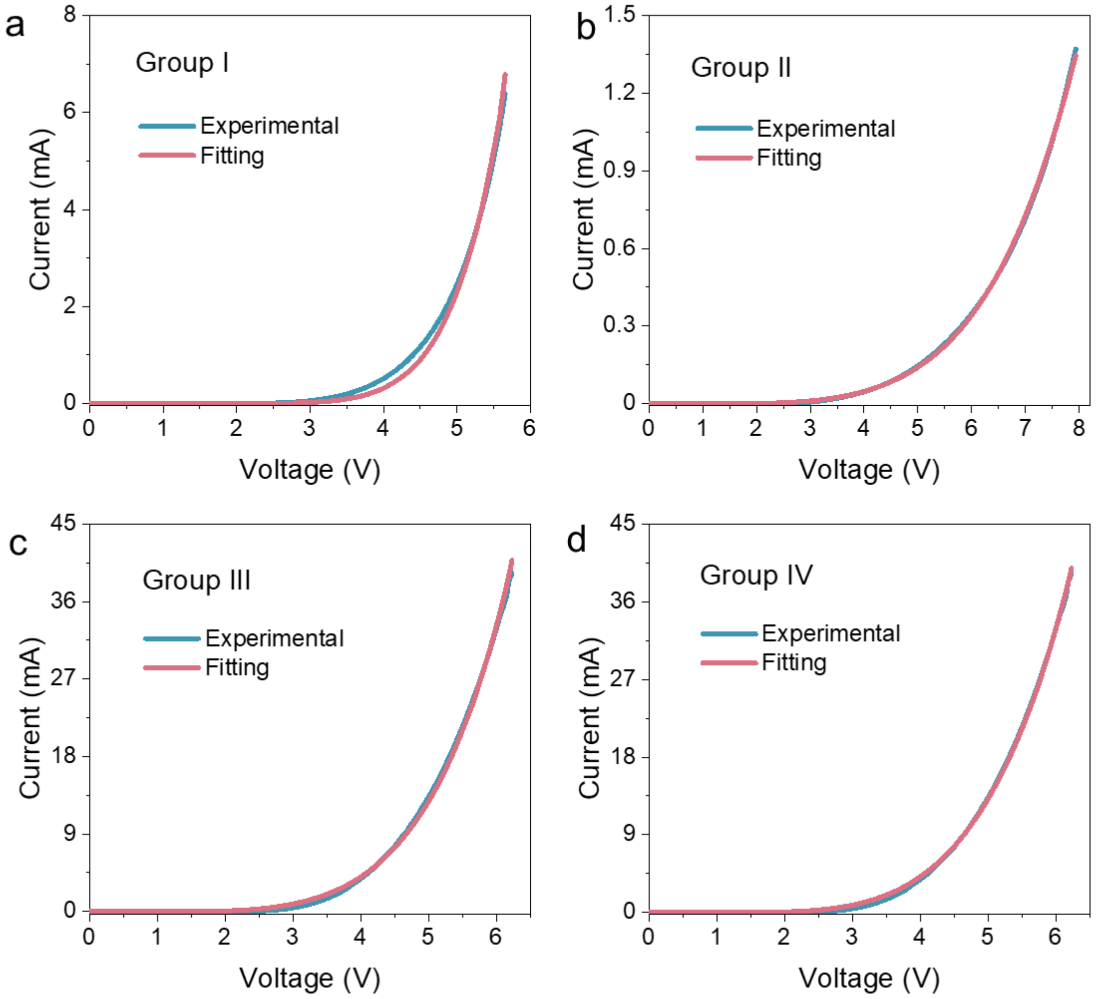

Figure S9. Numerical fitting results (red characteristics) and experimental data (black characteristics) for all device groups. **(a)** Group I, **(b)** Group II, **(c)** Group III, **(d)** Group IV.

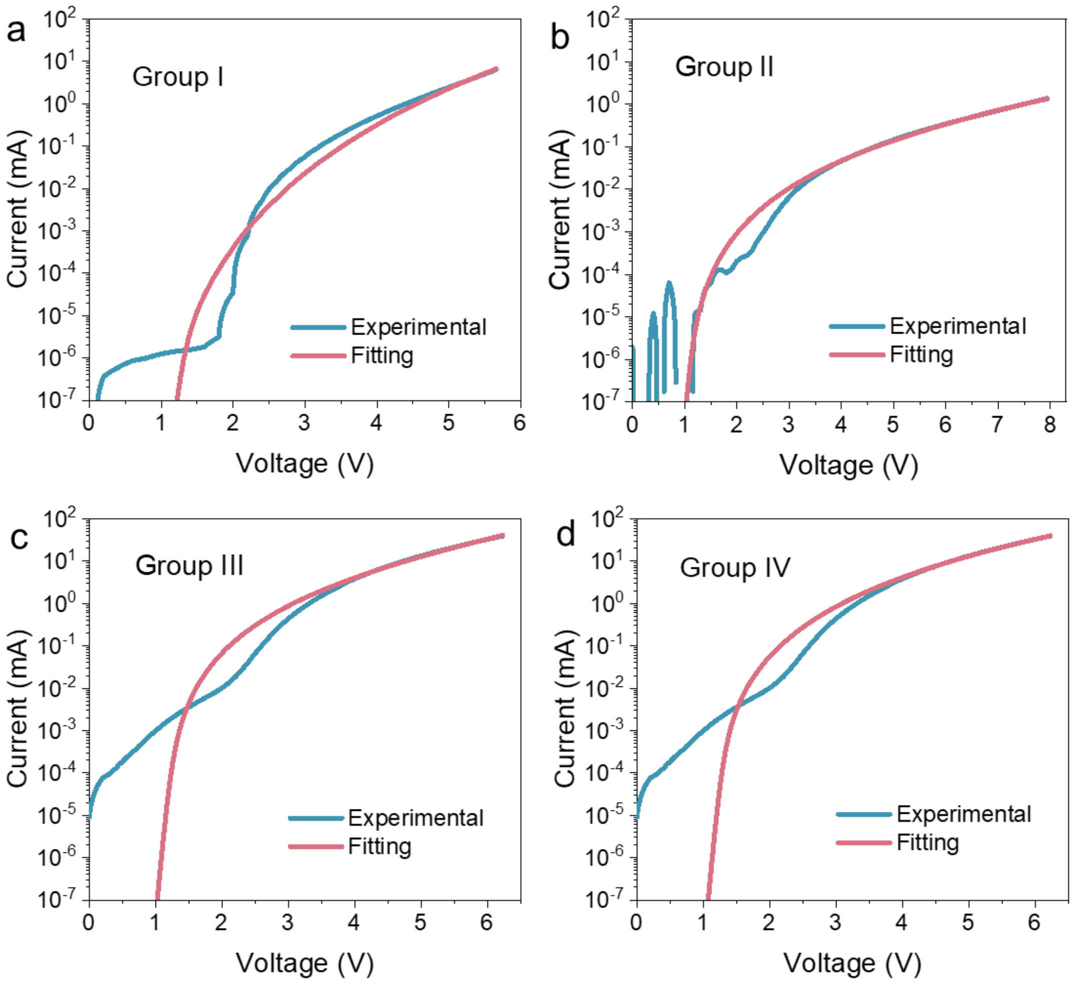

Figure S10. Numerical fitting results (red curves) and experimental data (black characteristics) for all device groups plotted on a logarithmic scale. (a) Group I, (b) group II, (c) group III, (d) group IV.

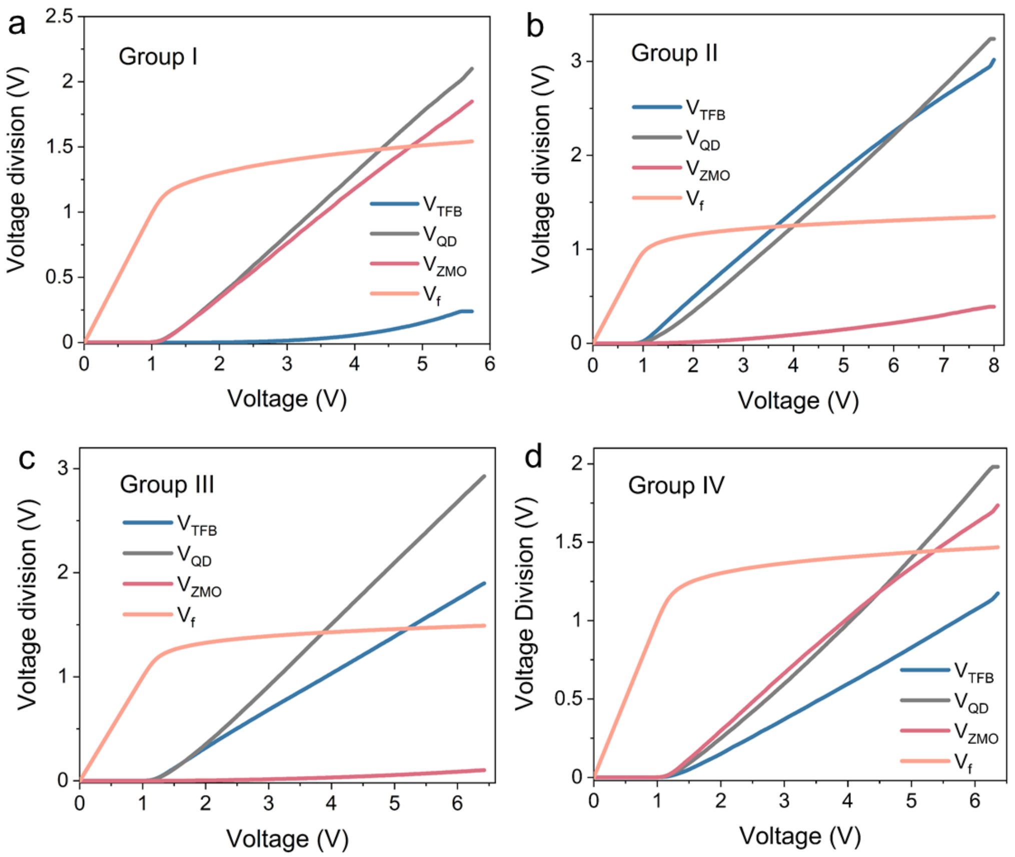

Figure S11. Voltage distribution of functional layers versus applied voltage for all device groups derived from numerical fitting. (a) Group I, **(b)** Group II, **(c)** Group III, **(d)** Group IV.

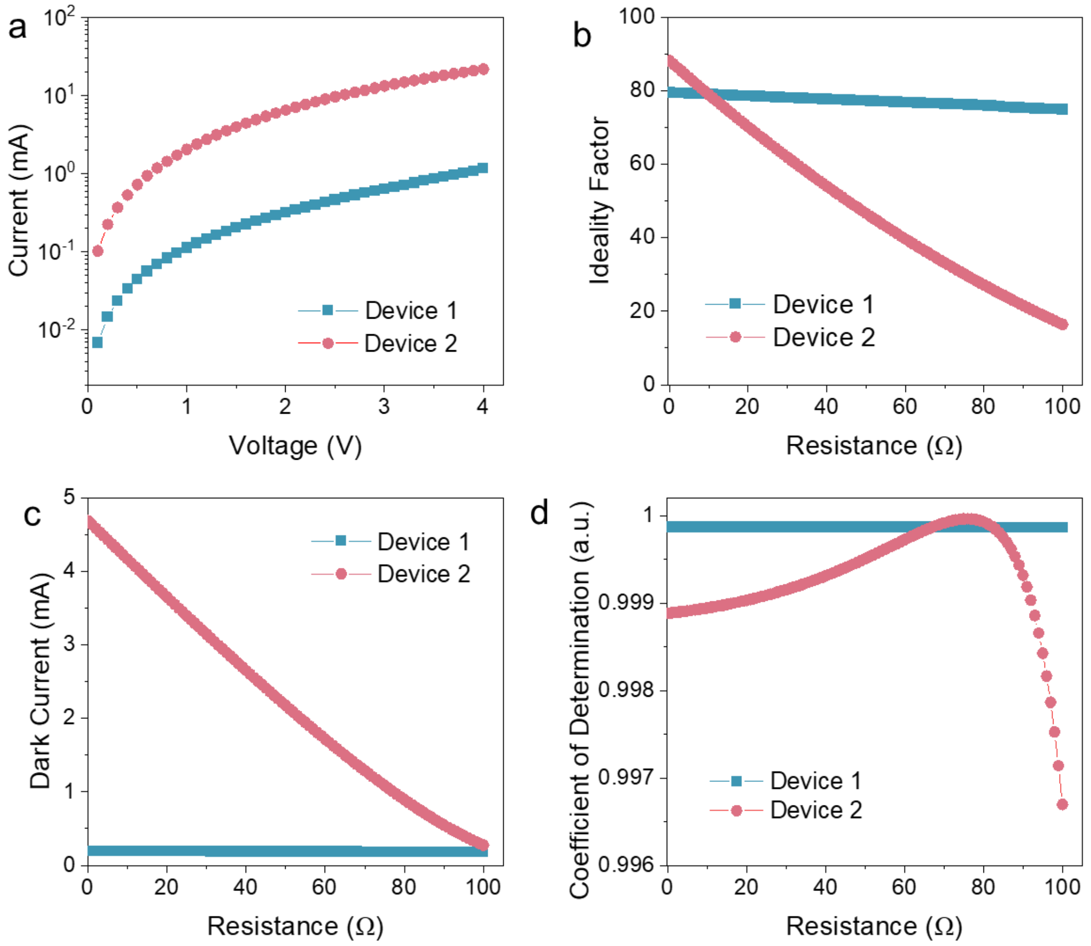

Figure S12. Schematic illustration of Rs impact on parameter extraction. (a) I-V characteristics of two representative devices; (b) Rs dependence of the extracted ideality factor; (c) Rs-induced variation in fitted saturation dark current (Is); (d) Rs influence on the coefficient of determination (R^2) for Schottky equation fitting.

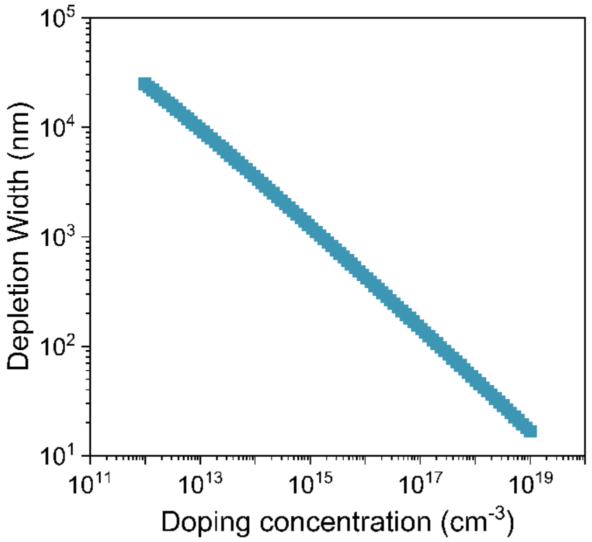

Figure S13. Doping concentration versus depletion layer width in conventional silicon-based PN junctions.

The relationship between the depletion layer width and doping concentrations for the silicon-based PN junction under zero bias, shown in Fig. S11, is calculated using the formula $W = \frac{2\varepsilon\varepsilon_0 V_{\mathrm{bi}}}{q}(\frac{1}{N_{\mathrm{A}}} + \frac{1}{N_{\mathrm{D}}})$, where $\varepsilon\varepsilon_0$, V_{bi}, N_{A}, and N_{D} represent the dielectric constant, built-in potential. Doping concentrations of the P-region and N-region, respectively. In Fig. S11, we assume that N_A=N_D. For a heterojunction PN junction, the formula for the depletion layer width is $W = \frac{2\varepsilon_1\varepsilon_2\varepsilon_0 V_{\mathrm{bi}}}{q(\varepsilon_2 N_{\mathrm{A}} + \varepsilon_1 N_{\mathrm{D}})}$. Based on the parameters of the functional layers in QLED devices (N_{A} or N_{D} $< 10^{13}$ cm^{-3}, ZMO relative dielectric constant ~ 8.3-12.7, QD relative dielectric constant ~ 6-8, TFB relative dielectric constant ~ 2-4, and V_{bi} ~ 1.9-2.2 V), the depletion layer width is estimated to be 5.61-8.06 μm for the TFB/QD heterojunction and 8.55-10.09 μm for the QD/ZMO heterojunction, both of which significantly exceed the actual thickness of QLED functional layers in practical devices.

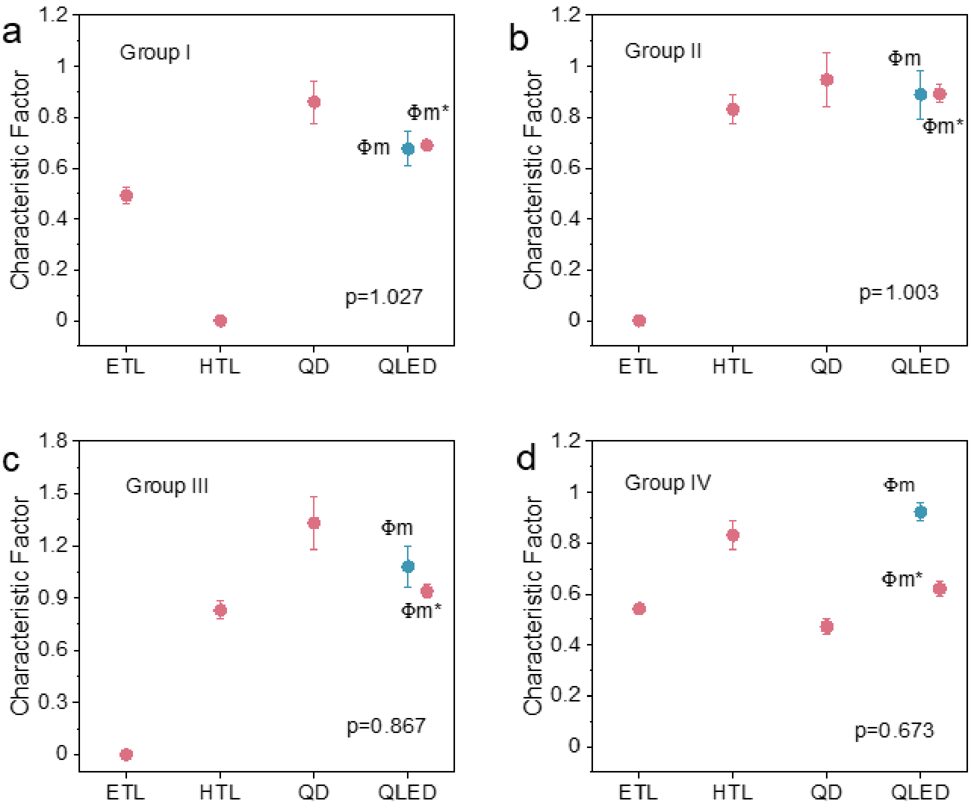

Figure S14. Fitting parameters of QLED devices and sub-devices with 95% confidence intervals for four groups. m_{HTL}, m_{QD}, and m_{ETL} represent characteristic factors of HTL, QD, and ETL layers, respectively, m^* represents predicted ideality factor; m represents fitted ideality factor from I-V characteristics.
